# *Drosophila* mRNA Localization During Later Development: Past, Present, and Future

**DOI:** 10.3389/fgene.2019.00135

**Published:** 2019-03-07

**Authors:** Sarah C. Hughes, Andrew J. Simmonds

**Affiliations:** ^1^Department of Medical Genetics, Faculty of Medicine and Dentistry, University of Alberta, Edmonton, AB, Canada; ^2^Department of Cell Biology, Faculty of Medicine and Dentistry, University of Alberta, Edmonton, AB, Canada

**Keywords:** *Drosophila melanogaster*, mRNA localization, organelle, neuronal differentiation, epithelial differentiation

## Abstract

Multiple mechanisms tightly regulate mRNAs during their transcription, translation, and degradation. Of these, the physical localization of mRNAs to specific cytoplasmic regions is relatively easy to detect; however, linking localization to functional regulatory roles has been more difficult to establish. Historically, *Drosophila melanogaster* is a highly effective model to identify localized mRNAs and has helped identify roles for this process by regulating various cell activities. The majority of the well-characterized functional roles for localizing mRNAs to sub-regions of the cytoplasm have come from the *Drosophila* oocyte and early syncytial embryo. At present, relatively few functional roles have been established for mRNA localization within the relatively smaller, differentiated somatic cell lineages characteristic of later development, beginning with the cellular blastoderm, and the multiple cell lineages that make up the gastrulating embryo, larva, and adult. This review is divided into three parts—the first outlines past evidence for cytoplasmic mRNA localization affecting aspects of cellular activity post-blastoderm development in *Drosophila*. The majority of these known examples come from highly polarized cell lineages such as differentiating neurons. The second part considers the present state of affairs where we now know that many, if not most mRNAs are localized to discrete cytoplasmic regions in one or more somatic cell lineages of cellularized embryos, larvae or adults. Assuming that the phenomenon of cytoplasmic mRNA localization represents an underlying functional activity, and correlation with the encoded proteins suggests that mRNA localization is involved in far more than neuronal differentiation. Thus, it seems highly likely that past-identified examples represent only a small fraction of localization-based mRNA regulation in somatic cells. The last part highlights recent technological advances that now provide an opportunity for probing the role of mRNA localization in *Drosophila*, moving beyond cataloging the diversity of localized mRNAs to a similar understanding of how localization affects mRNA activity.

## Introduction

Following transcription, mRNAs are regulated at multiple points during their lifetime. This begins in the nucleus where mRNAs undergo selective pre-mRNA splicing, base modification, sequence editing, and directed transport from the nucleus (reviewed in Stapleton et al., 2006; Maas, 2012; Rosenthal, 2015; Meier et al., 2016; Wei et al., 2017; Krestel and Meier, 2018; Schmid and Jensen, 2018; Wegener and Müller-Mcnicoll, 2018). Once exported to the cytoplasm, it is not at all guaranteed that mRNAs will be translated, as many are sequestered away from ribosomes in a non-translating pool (Patel et al., 2016; Standart and Weil, 2018). In terms of the mechanisms that regulate mRNAs in the cytoplasm, microRNAs (miRNAs), RNA interference (RNAi), and similar pathways are relatively well-characterized (Chandra et al., 2017; Noh et al., 2018). Regulated mRNA transcripts are often associated with cytoplasmic ribonucleoprotein complexes (RNPs), that can regulate translation, e.g., Stress Granules (Buchan and Parker, 2009) or degradation (Towler and Newbury, 2018) or both, e.g., RNA processing (P)-bodies (Standart and Weil, 2018). It is becoming clear that there are multiple examples of mRNA regulation at the level of translation via direct regulation of ribosome binding or processivity as (reviewed in Abaza and Gebauer, 2008). All of these different mRNA regulatory pathways are found in *Drosophila melanogaster*. Among these, the phenomenon of directed cytoplasmic restriction of mRNA localization is commonly observed and has been studied during oogenesis and in the early embryo. However, the underlying roles for this process, beyond the formation of the cellularized-blastoderm, remains poorly understood.

### Identifying Subcellular mRNA Localization in *Drosophila*

Much of the initial study of mRNA localization in the cytoplasm of *Drosophila* cells was driven by direct observation of transcript location. Visualization of specific mRNAs first became possible with the adaptation of *in–situ* hybridization (ISH) techniques for *Drosophila* where anti-sense probes (either DNA or RNA) hybridize to mRNA targets in fixed cells or tissues (Singer and Ward, 1982). The first examples of detection of RNAs in fly cells used radiolabeled anti-sense ISH probes on sectioned ovaries or late-stage embryos (Brennan et al., 1982; Hafen et al., 1983; Levine et al., 1983). Non-radioactive methods followed using digoxigenin, biotin, or other hapten UTP conjugates to synthesize ISH probes recognized by antibodies conjugated to alkaline phosphatase or peroxidase (Tautz and Pfeifle, 1989; O'neill and Bier, 1994). The development of practical methodologies for fluorescent *in-situ* hybridization (FISH) for *Drosophila* tissues expanded the utility of ISH allowing visualization and three-dimensional spatial reconstruction of mRNA localization within the cell by confocal microscopy (Hughes et al., 1996; Hughes and Krause, 1998, 1999). Later enhancements to FISH protocols, including signal amplification techniques, provided brighter signals facilitating high-throughput screens (Lécuyer et al., 2008; Wilk et al., 2010; Jandura et al., 2017). The utility of FISH was extended again with the development of single molecule (sm) FISH which allows an approximate detection at the resolution of a single mRNA (Femino et al., 2003). Recently smFISH has been successfully adapted to *Drosophila* cells and tissues (Bayer et al., 2015; Little and Gregor, 2018; Titlow et al., 2018).

However, our ability to observe the phenomenon of mRNA localization has traditionally exceeded our ability to probe the functional role in regulating the mRNA, in terms of translation or stability. Two types of cells feature prominently in past studies of mRNA localization in *Drosophila*. The first is the oocyte, which develops as a cyst of 16 germline cells, surrounded by epithelium consisting of somatic follicle cells. The second is the fertilized embryo, a coenocyte with multiple nuclei until 2:10 h of development, when membranes enclose individual nuclei into individual cells forming a cellularized blastoderm. In the syncytial embryo the best functionally understood example for mRNA localization is that of anterior *bicoid* (*bcd*), that helps establish body polarity, although there are many other known roles in the early embryo and germ cells, (reviewed in Cho et al., 2006; Lasko, 2011, 2012; Weil, 2014, 2015; Laver et al., 2015; Yamashita, 2018).

The widespread prevalence of examples of localized mRNA regulation events in oocytes and the early syncytial embryo prompts two alternative viewpoints. Either the nature of egg formation and syncytial development has been selected for these events, or cytoplasmic mRNA localization is a widespread event in all cell types and was merely easier to detect in relatively large cells like the early embryo.The preponderance of examples of localzied mRNAs and conserved functional requirements for mRNA regulatory proteins during later development suggests that the latter scenario is more likely. Proteins known to regulate mRNA localization in early *Drosophila* embryo development (e.g., Staufen, Stau) are conserved in metazoans, reviewed in Heraud-Farlow and Kiebler (2014) and Piccolo et al. (2014) or are required in somatic lineages such as neuroblasts that form post-cellularization (St. Johnston et al., 1991; Li et al., 1997; Matsuzaki et al., 1998). Similarly, some mRNAs, localized in germ cells or the early embryo such as: *Cyclin B, oo18 RNA-binding protein, Protein kinase, cAMP-dependent, catalytic subunit 1, nanos* (*nos*), or *Heat Shock Protein 83* (*Hsp83*), are expressed during later development or conserved in organisms without a syncytial embryo (Raff et al., 1990; Gavis and Lehmann, 1992; Ding et al., 1993; Lantz and Schedl, 1994; Dubowy and Macdonald, 1998; Subramaniam and Seydoux, 1999; Tsuda et al., 2003). As described below, there has been some past evidence showing that mRNA localization is essential in regulating aspects of specific lineages such as differentiating neuroblasts (Knoblich et al., 1995; Broadus et al., 1998) and reviewed in Martin and Ephrussi (2009) and Medioni et al. (2012). Additional support for a more widespread role for mRNA localization during later development, which in this review refers to the somatic lineages formed post-cellular blastoderm, comes from ongoing FISH screens (Jambor et al., 2015; Wilk et al., 2016). These have now enumerated hundreds of mRNAs with specific localization patterns in a wide variety of cell lineages.

To outline the potential scope of mRNA regulation during later *Drosophila* development, we first describe the known examples of mRNA regulation during later Drosophila development. We then speculatively extrapolate potential roles for a large number of mRNAs, directly observed as subcellularly localized, during later *Drosophila* development. Finally, we highlight new methods that promise to enable the future determination of the functional roles for subcellular mRNA localization in the smaller, somatic cells that form the various tissues of the post-blastula embryo, larvae, and the adult.

## Past Identified Roles for mRNA Localization During Later *Drosophila* Development

Currently, the most well-characterized examples of functional roles for localized mRNAs during later *Drosophila* development, come from highly polarized cells such as neurons and epithelia. Like the oocyte and early embryo, the morphology of these cells is highly polarized, and likely facilitates observation of subcellular localization.

### Localized mRNAs Direct Neural Stem Cell Differentiation

Embryonic neuroblasts (NBs) are neural stem cells that delaminate stereotypically from the ventral nerve cord during later (stage 9) embryonic development (Hartenstein and Campos-Ortega, 1984). NBs divide asymmetrically from stages 9 to 11 with one self-renewing daughter, and a smaller daughter called a ganglion mother cell (GMC). GMCs differentiate at stage 13 into neuronal and glial lineages. During late embryogenesis, a portion of the NBs become quiescent and then during early larval stages neuroblasts re-enter the cycle and begin the second wave of neurogenesis undergoing multiple rounds of asymmetric cell divisions exiting the cell cycle in pupal stages (Homem and Knoblich, 2012). In these cells, mRNA localization is coupled with cell division to direct asymmetric inheritance of transcription factors directing differentiation.

The b*azooka* (*baz*) mRNA encodes the *Drosophila* Par-3 homolog and is localized to an apical cytoplasmic crescent in embryonic NBs, reviewed in Homem and Knoblich (2012). Baz protein is also localized in an apical crescent, but specifically in metaphase NBs. Apical Baz is required for proper orientation of the spindle in mitotic NB cells, and localization failure leads to misorientation of the spindle relative to the apical/basal pole, resulting in mispositioning of the GMCs and defects in a portion of GMC fates (Kuchinke et al., 1998). Prospero protein is asymmetrically localized in NBs and is portioned to the GMCs (Hirata et al., 1995; Knoblich et al., 1995). The *prospero* (*pros*) mRNA encoding a transcription factor that defines GMC identity is asymmetrically localized, initially at the apical cortex and then to the basal cell cortex during NB cell division (Broadus et al., 1998). The localization of *baz* and *pros* requires Stau and Inscuteable (Insc). Stau binds the *pros* mRNA 3′UTR directly. Binding of Stau is required for the basal localization of *pros* mRNA, but not Pros protein (Broadus et al., 1998). Stau localizes to an apical crescent in interphase NB cells, but during mitosis, Stau is found at the basal cortex. Another basally localized protein, Miranda (Mira), is also required for both Pros protein and *pros* mRNA localization via interaction with Stau (Schuldt et al., 1998). Insc regulates *pros* mRNA relocalization from the apical to the basal cortex in late interphase to prophase cells (Li et al., 1997). Notably, *Insc* mRNA is cortical during interphase yet is found throughout the cytoplasm during mitosis, whereas Insc protein is always localized at the apical cortex of NB cells. In embryonic NBs, Egalitarian (Egl) is required for *Insc* localization (Mach and Lehmann, 1997). Egl, Bicaudal-D (Bic-D) and the Dynein transport complex function during oogenesis and embryogenesis and in embryonic NBs to localize *Insc* mRNA (Hughes et al., 2004). Other *Insc* regulators have also been identified in NBs, including DEAD-box RNA dependent ATPases that control many aspects of RNA metabolism (reviewed in Putnam and Jankowsky, 2013) and Abstrakt (Abs) required for translation of Insc protein but not for *Insc* mRNA localization in embryonic neural stem cells or NBs (Irion et al., 2004). Ultimately, despite a relatively well-developed mechanistic knowledge of how *pros* mRNA is localized, the functional role for this localization remains unclear as *pros* mRNA and protein are localized independently, and the two pathways may redundantly direct GMC fate (Broadus et al., 1998).

Localization of mRNAs and their encoded proteins are also required for establishing NB polarity during larval neural differentiation. Subcellular localization of *mira* mRNA is required for this process (Bertrand et al., 1998). Using a combination of a MS2 RNA labeling system and nanobody expression to detect protein, misdirection of *mira* mRNA to nuclear, apical or basal regions, identified two pools of *mira* mRNA during mitosis (Ramat et al., 2017). One pool localized to the spindle, and the other localized at the basal pole of the NB. When *mira* mRNA was directed away from the basal pole, there were defects in mitosis (Ramat et al., 2017). Mira protein is co-localized to the basal pole via direct interaction with *mira* mRNA either directly or through recruitment of further factors. This effect is reminiscent of how *oskar* (*osk*) mRNA localization in the oocyte is required for localized translation of the Oskar protein which is then required to maintain *osk* mRNA localization (Rongo et al., 1995).

Subcellular mRNA localization directs several aspects of embryonic and larval NBs by supporting the establishment of polarity that is essential for NB self-renewal and correct differentiation of GMCs into neurons or glia. The same process is also essential for the correct differentiation of the adult nervous system. As the investigation into the molecular machinery required for NB polarity and asymmetric division continues, it is likely that additional contributions of mRNA localization will be identified as essential. For example, mRNA localization events could regulate how proteins interact with the actomyosin skeleton to direct spindle orientation, which is required for proper NB division and differentiation. New protein players in these processes (e.g., Moesin, Moe) have been identified (Abeysundara et al., 2018), but a similar role of localization of the encoding mRNAs in these processes have not yet been examined.

## Localized mRNAs Regulate Development and Plasticity of Dendrites and Axons

Neuronal axons can be extremely long, and in many different organisms localized mRNAs have been identified, that regulate corresponding local translation of protein production (reviewed in Rodriguez-Boulan and Powell, 1992; Piper and Holt, 2004; Yoo et al., 2010; Jung et al., 2012; Sahoo et al., 2018). Local translation is thought to facilitate rapid cellular response for events like neuronal circuit-based local remodeling of dendrites and synapse numbers, as the time it takes mRNAs to emerge from the nucleus, would drastically slow down a remodeling response (Medioni et al., 2012). Previous *ex vivo* and *in vivo* studies in growing *Xenopus* and mouse axons have demonstrated a clear link between axonal mRNA localization, local translation and the direction of axon growth (Medioni et al., 2012). Remodeling of the neurons in terms of pruning, regrowth and branching of axons is required for the refinement of neural circuits governing larval and adult behaviors (Medioni et al., 2012). While it has been assumed that localized mRNA regulating local protein translation are also conserved in *Drosophila* axons and dendrites, to date there have been relatively few studies confirming this, some of which are highlighted below (Macdonald and Struhl, 1988; Brechbiel and Gavis, 2008; Misra et al., 2016) reviewed in (Rodriguez-Boulan and Powell, 1992).

One *Drosophila* cell type, where spatiotemporal mRNA localization has been shown to regulate changes in differentiated axons, is the mushroom body γ neurons of the larval brain. Mushroom bodies play a role in olfactory learning and memory (Heisenberg et al., 1985; Heisenberg, 2003). During larval development and pupal metamorphosis, mushroom body axonal branches are pruned selectively. These subsequently regrow to form adult specific branches (Lee et al., 1999; Watts et al., 2003). IGF-II mRNA-binding protein (Imp) was identified by mutagenesis as important for axonal remodeling or regrowth of axons that have been pruned, but not in their initial axon growth (Medioni et al., 2014). Using live imaging of pupal brains, it was observed that GFP–Imp is localized to specific RNP particles that move actively via microtubule-dependent transport within axons undergoing remodeling (Medioni et al., 2014). Imp selectively associates with the 3′UTR of *chickadee* (*chic*) mRNA (encoding the fly Profilin homolog) which localizes to growing γ neurites (Medioni et al., 2014).

The translational repressor proteins Nos and Pumilio (Pum) are required for germline development and establishing abdominal polarity in the early embryo (Asaoka-Taguchi et al., 1999; Cho et al., 2006). Gain and loss of function studies during later development show that both Nos and Pum are required to regulate dendrite branching in specific subsets of larval dendritic arborization (da) neurons including Class IV (not class I or II) in the peripheral nervous system (Ye et al., 2004). The shape, branching patterns and growth of the dendrites are correlated with the activity of the neuron. During development, these neurons undergo morphogenesis to form extensive arborization trees, providing easily observed phenotypes and thus, are used extensively for forward-genetic screens in flies. Direct imaging showed that *nos* mRNA is localized not only in the cell body but also in RNPs which are distributed along the dendrite and axon processes of class IV da neurons in a process mediated by recognition of sequences in the 3′ UTR (Brechbiel and Gavis, 2008). Live cell imaging of the *nos* mRNA showed that dynein machinery components are required for transport of *nos* RNP particles in the dendrites (Xu et al., 2013). Also, RBPs Rumpelstiltskin (Rump) and Osk, known to be required for localization of *nos* mRNAs in oocytes, are also required in the formation and transport of *nos* RNP particles in dendrites (Xu et al., 2013).

These known examples confirm that mRNA localization is required in both early development as well as later, during morphogenesis of differentiated neurons. Intriguingly, many of the localized mRNAs and their localization factors appear to be the same in these two systems. Supporting the conclusion that a common regulatory strategy may be shared between early development and neurogenesis, an RNA interference screen for RNA regulatory proteins that affects dendrite morphogenesis in Class IV da neurons identified some proteins and a translation factor previously shown to regulate maternal mRNA localization in embryos and oocytes (Olesnicky et al., 2014). Further investigation into which and how mRNAs are implicated in the dendrite morphogenesis will be a future area of interest and study.

Most of the currently known examples of localized mRNA translation in *Drosophila* neurons largely mirror those in mammals supporting an assumption that these events are conserved (reviewed in Medioni et al., 2012). The localization of mRNAs is essential for the proper axon guidance, formation and remodeling of dendrites to form neural circuits throughout development, as discussed above. The proper localization of mRNAs are also required for memory and learning in both flies and humans (reviewed in Greenspan, 2003; Agnès and Perron, 2004; Puthanveettil, 2013; Olesnicky and Wright, 2018). However, genetic screens in flies are starting to identify additional functional roles for proteins that likely have a role in mRNA localization (for example Song et al., 2007; Martin and Ephrussi, 2009; Hayashi et al., 2014; Misra et al., 2016).

## Localized mRNAs are Required at Neuromuscular Junctions

The neuromuscular junction (NMJ) is a highly specialized region where motor neurons synapse to specific muscle targets (Menon et al., 2013). Formation of new synapses is required during early neuronal development, and synapse growth requires targeting of specific mRNAs to the NMJ in addition to the localized recruitment of proteins and organelles (Medioni et al., 2012). It is also thought that the localized translation of mRNAs underlies plasticity at synapses (Kindler and Kreienkamp, 2012; reviewed in Jung et al., 2012). *Drosophila* larval NMJs have emerged as a powerful *in vivo* model to study the role of localized mRNAs and localized translation in synaptic development and plasticity. In *Drosophila* larvae there are 32 motor neurons per abdominal hemisegment, and the NMJ is quite large and easily imaged. Larval NMJs are composed of structures called synaptic boutons that are arranged like beads on a string and exhibit developmental and functional plasticity while being stereotypically organized (Keshishian et al., 1996).

Localized mRNAs and localized translation of mRNAs in the motor neuron or NMJ are required for both the development of synapses and the plasticity of the NMJ presynaptically and post-synaptically. This is mediated by RNPs that are transported along neuronal processes in response to stimuli or development. How RNPs reach the correct location at the NMJ after exiting the nucleus remains an open question. Several groups have shown that RNPs generally move on dynein or kinesin motors and there are also some studies that implicate actin filaments or actin-based motors (Doyle and Kiebler, 2011; Medioni et al., 2012). Studies using genetic or proteomic approaches have identified some mRNA targets and RNA binding proteins at the NMJ (for example Raut et al., 2017) and reviewed in Hörnberg and Holt (2013), but there are likely many more required in this dynamic structure.

The fly NMJ has also been informative in understanding the underlying mechanisms required for localizing mRNAs in neurons including the role of the actin cytoskeleton (Packard et al., 2015). The actin-binding protein Muscle-specific protein 300 kDa (Msp300, also known as Syne1) is required to localize specific mRNAs post-synaptically. mRNAs including *par-6* and *Magi* mRNA are enriched at the postsynaptic region of the NMJ while others such as *discs large 1* (*dlg1*) are not. In *Msp300* mutants, there is a loss of localization of *par-6* and *magi* but not *dlg1*. This is due to defective transport of the *par-6* and *magi* mRNAs as opposed to a defect in export from the nucleus or stability of the mRNA transcripts (Packard et al., 2015). Msp300 was demonstrated to be required for maturation of the synaptic boutons. Msp300 protein is organized into long striated filaments termed “railroad” tracks that extend from the nucleus to the edge of the NMJ (Packard et al., 2015). This organization is thought to work in conjunction with an unconventional myosin motor protein Myosin 31DF (Myo31DF) for proper localization of these postsynaptic mRNAs (Packard et al., 2015).

Similar to that which occurs in neurons and neuronal stem cells, the *Drosophila* NMJ represents an excellent example of the conservation of mRNA localization events between human and *Drosophila* (Vazquez-Pianzola and Suter, 2012). Additionally, the mRNA localization events in NMJs repeat themes from earlier developmental stages where localized proteins and mRNA targets the functioning of oocytes, and early embryogenesis are also active during later developmental stages. Again, because of its relatively large size and polarized morphology, the *Drosophila* NMJ is an elegant, easily visualized, and genetically amenable system by which both pre- and postsynaptic roles of localized mRNA an RNA binding proteins can be analyzed. In summary, localization of mRNAs or RNA binding proteins is an essential part of many aspects of neuronal differentiation and function during later *Drosophila* development. While many localized mRNAs with localized translation are known, the molecular mechanisms related to the role of this local translation or the mechanisms that recruits the mRNA to specific cell domains, have yet to be discerned. Further investigation into the specific localization and function of these players, should provide further insights into the formation and plasticity of the neuronal system.

## Known Roles for mRNA Localization in Epithelial Cell Lineages

The role of mRNA localization in later *Drosophila* development is far less characterized in cell types other than neurons. The numerous examples of neuronal mRNA localization may be an overrepresentation and may reflect critical morphological features of highly polarized neurons and neuronal stem cells, such as large size and polarization which facilitate the discovery of localized mRNAs. Fascinatingly, many protein regulating mRNAs in oocytes, early embryos and neurons are expressed in multiple lineages during later development, (Brown and Celniker, 2015) making it likely that mRNA targets are regulated by cytoplasmic localization in other cell lineages that compose the majority of gastrulating embryos, larvae, and adults.

### Localized mRNAs Encoding Proteins Involved in Establishing Epithelial Cell Polarity

Many other proteins involved in establishing apical/basal polarity in epithelial cells have localized mRNAs. Cell junctions are multi-protein structures localized to the apical-lateral or lateral membrane that are best characterized in epithelial cell lineages (Tepass et al., 2001; Cavey and Lecuit, 2009; Tepass, 2012). Atypical protein kinase C (aPKC), Crumbs (Crb), Stardust (Sdt), Baz, and Patj help establish the apical plasma membrane domain and have been shown to interact directly in various cells of epithelial lineage (Tepass et al., 1990; Bhat et al., 1999; Bachmann et al., 2001; Hong et al., 2001; Médina et al., 2002; Nam and Choi, 2003; Hutterer et al., 2004; Sen et al., 2015). Similar to what occurs in neural stem cells, *baz* mRNA is restricted to a narrow apical domain in the cytoplasm of epithelial cells in late-stage embryos (Kuchinke et al., 1998). A similar pattern of apical mRNA localization was observed with *sdt* mRNA. The mechanism of this apical transport of *sdt* mRNA includes alternative splicing of *sdt* to include an exon which directs apical transport in a dynein-dependent manner (Horne-Badovinac and Bilder, 2008). Notably, a dynein-dependent mechanism also targets the *crb* mRNA to the apical region of the epithelial-lineage somatic cells (follicle cells) that surround the developing oocyte (Li et al., 2008). For polarized epithelial cells, mRNA localization does seem to have a functional role. Work using mammalian cells also suggests that there may be specialized regulation centers that co-regulate mRNAs that are encoding junctional proteins. Recently, a role for localized translation of collections of mRNAs was restricted to small cytoplasmic regions above nascent adhesion sites in mammalian amoeboid cell lineages. These were termed spreading initiation centers (SICs) (Bergeman et al., 2016). It will be particularly interesting to see if there is similar co-regulation of mRNAs encoding adhesion complex proteins in *Drosophila* embryo and larval cells and if these events are conserved in other organisms.

## Apical Localization of mRNAs Encoding Secreted Proteins

mRNAs encoding secreted proteins are directed to the ER by both translation dependent signal peptide-mediated and translation-independent pathways (reviewed in Hermesh and Jansen, 2013; Cui and Palazzo, 2014). However, there is evidence that mRNA localization is critical for regulating signaling events between epithelial cells, independently of SRP-mediated trafficking to the ER. In *Drosophila* epithelial cells, the mRNA encoding *wingless* (*wg*) is directed to the region just under the apical plasma membrane, within the cytoplasm of ectodermal cells, in stage 4–6 embryos. This mRNA localization is required for the production of an active Wg signaling protein (Simmonds et al., 2001). Notably, *wg* mRNAs are associated with punctate cytoplasmic RNP particles that are transported to the apical cytoplasm in a dynein-dependent mechanism (Wilkie and Davis, 2001; Najand and Simmonds, 2007). The cis-acting signals for *wg* mRNA localization and anchoring are found within the 3′UTR of the mRNA directing aggregation of multiple *wg* mRNAs, which appears as discrete cytoplasmic foci (Simmonds et al., 2001; Najand and Simmonds, 2007; Dos Santos et al., 2008). The regulation of translation and localization of *wg* mRNA are not linked directly as non-translatable *wg* mRNAs, and reporter genes fused to the *wg* 3′UTR are localized equally, as well as mRNAs with an intact open reading frame (Simmonds et al., 2001; Najand and Simmonds, 2007). The requirement for apical localization of the *wg* mRNA also calls into question where the translated protein enters the ER/Golgi complex for secretion. There are examples of apically localized sub-regions of the ER in highly polarized cells that also have multiple examples of localized mRNAs such as *Drosophila* neuroblasts (Smyth et al., 2015; Eritano et al., 2017), but the coincidence of *wg* mRNA and specialized ER domains have not yet been studied. Thus, similar to what has been shown for neurons, there is evidence that mRNA localization has a functional role in polarized epithelia in *Drosophila* embryos after they cellularize. However, how these mRNA localization events regulate the encoded proteins remains mostly elusive.

### The Present State of Affairs: There Are Many Different Localized mRNAs in Many Different Cell Lineages During Later *Drosophila* Development

Based on the few known examples, the roles of mRNA localization have been found in most of the somatic cells that make up the gastrulating embryo and were not considered to be that prevalent in larval and adult tissues. However, in the past few years, the number of known localized mRNAs in later development has increased significantly. Systematic screens have identified localized mRNAs in numerous embryo somatic cell lineages, larval gut, imaginal discs, salivary glands and adults, which has significantly changed how mRNA localization is viewed in terms of *Drosophila* development. Firstly, most mRNAs manifest some pattern of subcellular localization in one or more cell lineages. Secondly, localized mRNAs encode a wide variety of proteins with diverse functions, far more than those few that have been previously characterized.

## Determining the Extent of mRNAs Localization During Later *Drosophila* Development

The advent of aptamer tags based on specific mRNA hairpin motifs facilitated the tracking of mRNAs in live cells (reviewed in Weigand and Suess, 2009). Different RNA aptamers recruit specific binding proteins, which then fuse to fluorescent proteins that demark localization of tagged mRNA within the cytoplasm. The most common of these is MS2 tagging, using an RNA motif bound by the MS2 coat protein (Bertrand et al., 1998), reviewed in (Heinrich et al., 2017a). A transgene encoding MS2 coat-protein GFP, fused to a nuclear localization signal, is co-expressed in cells with MS2 tagged GFP. Transgenes expressing mRNAs with multiple MS2 aptamers (e.g., 24x) that recruit MS2-GFP, prevent it from entering the nucleus and marks the tagged mRNAs. These techniques have been adapted for *Drosophila* (reviewed in Abbaszadeh and Gavis, 2016) and have facilitated screening based on direct observation of mRNA localization in live neurons. Misra *et al*. recently performed such a screen in Class IV da neurons using semi-random transposon insertion of an MS2 RNA aptamer into the genome to track the encoded mRNAs. 541 lines were screened, and 47 genes had transcripts that are subcellularly enriched in class IV da neuron processes (Misra et al., 2016). Many of the encoded proteins were previously associated with subcellularly localized mRNAs including *CG9922, coracle (cora), fatty acid binding protein (fabp), scheggia (sea), High mobility group protein D (HmgD), and schnurri (shn*) (Misra et al., 2016).

**Figure 1:**
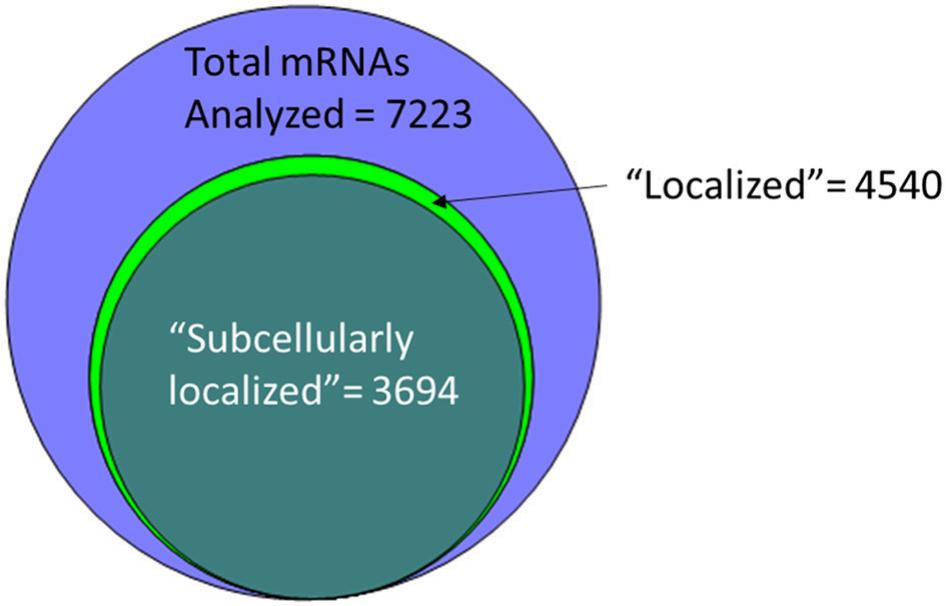
More than half of the mRNAs analyzed by low-resolution FISH in post-syncytial (Stage 4+) embryos; larva or adult cells are annotated as having subcellular localization (Wilk et al., 2016).

An alternative to insertional screens is direct observation of mRNA localization by ISH. In the past few years, several groups have provided a significant resource to the fly community via large-scale FISH screens that assay the localization of thousands of different mRNAs in cells of late-stage embryos, larvae and adults (Olesnicky et al., 2014; Jambor et al., 2015; Wilk et al., 2016; Zhang et al., 2017). Many of these are publicly available in the searchable Fly-FISH database (http://fly-fish.ccbr.utoronto.ca/) and the Dresden Ovary Table (DOT) database (http://tomancak-srv1.mpi-cbg.de/DOT/main.html) (Wilk et al., 2013, 2016; Jambor et al., 2015). Of particular interest, 167 mRNAs have different localization patterns in different cell types or at different times during development, suggesting that localization is dynamic and is cell lineage dependent (Wilk et al., 2010). Examination of this relatively unbiased screening data suggests that rather than being a rare event, at least half of the mRNAs are restricted in their distribution within the cell. For example, the Fly-FISH database reports localization data including approximately 6,800 mRNAs expressed in post-syncytial (past stage 4) embryos, and larval tissues via low-magnification FISH images (Wilk et al., 2010). Of these, 3509 (52%) are annotated as having a subcellular localization pattern in embryonic or larval tissues (Wilk et al., 2016) (Figure 1). Notably, one of the most commonly annotated patterns of localization was “cytoplasmic foci” (Wilk et al., 2016), which may suggest incorporation into one or more cytoplasmic RNPs or organelles. The localization data from these screens as well as examples curated from the literature has been collated in searchable databases such as RNALocate (http://www.rna-society.org/rnalocate), (Zhang et al., 2017). However, the precise number of unique localized mRNAs is hard to determine, as different groups use variable language and ambiguous or non-standard gene annotations.

### Many Localized mRNAs Cluster by Protein Function

**Figure 2:**
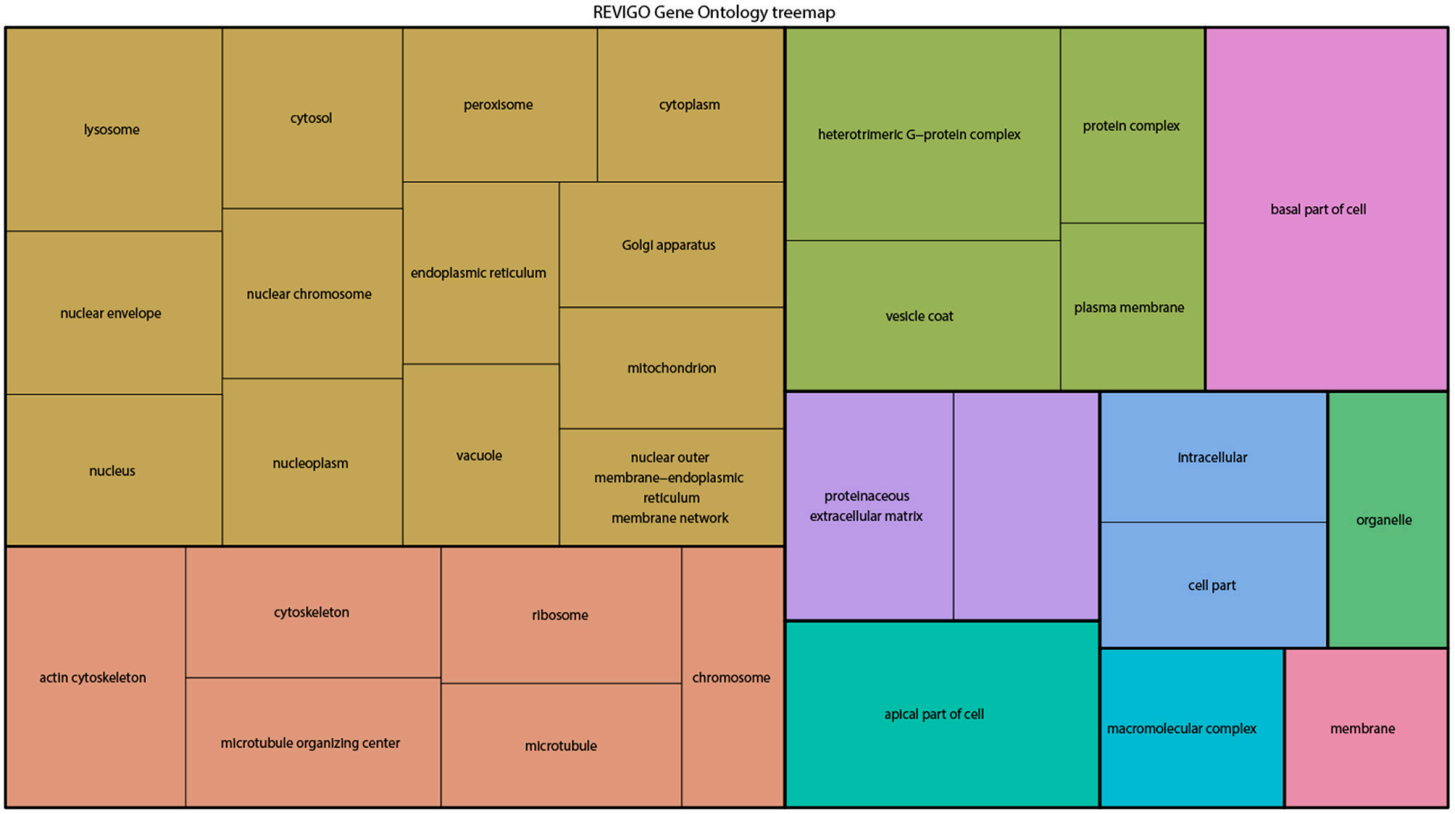
A ReviGO treemap showing the relative appearance of GO terms for the proteins encoded by3549 unique mRNAs annotated as localized in post-syncytial stage *Drosophila* embryos (2:10+), larval tissues and adult follicle cells ranked by fold-enrichment compared to the number of times the GO term is used for the entire genome (PANTHER). Common colors represent groupings based on parent GO terms, and each rectangle is proportional to the relative enrichment of the GO term compared to the whole genome.

From the above databases, 4049 unique genes are annotated as “subcellularly localized” in cells in post syncytial embryos (stage 4+), larva and adults. To define the scope of mRNA localization during later development, commonalities of the proteins encoded by localized mRNAs were identified. To disambiguate differences in gene names reported by different screens, mRNA lists were validated using the “ID converter” function of FlyBase (http://flybase.org/convert/id) (Gramates et al., 2017). Non-protein-coding genes were eliminated from the combined list and ambiguous gene names corrected manually. The resulting list of 3549 unique genes was clustered by gene ontology (GO) terms based on the “cellular component” of the proteins encoded by each (Ashburner et al., 2000; Tweedie et al., 2009; Consortium, 2017). A PANTHER Overrepresentation Test (version 13.1) identified GO terms that were enriched by proteins encoded by localized mRNAs, compared to their frequency in the whole genome (Table 1). The proportional overrepresentation of GO terms relative to the whole genome was visualized using REVIGO (Figure 2) (Supek et al., 2011).

**Table 1:** PANTHER analysis of enrichment of GO terms for 3549 mRNAs shown to be localized post-syncytial-stage (+2:10) of embryo development compared to the distribution of the same GO terms over the entire *Drosophila* genome.

**Analysis Type:**	**PANTHER overrepresentation test (Released 2017-12-05)**						
**Analyzed List:**	**Localized mRNAs (*****Drosophila melanogaster*****)**						
**Reference List:**	***Drosophila melanogaster*** **(all genes in database)**						
**Test Type:**	**FISHER**						
**PANTHER GO-Slim cellular component**	**Total GO in** ***Drosophila***	**GO—localized mRNAs**	**Expected number**	**±**		***p*****-value**	**FDR**
apical part of cell (GO:0045177)	3	2	0.75	+	2.66	2.63E-01	4.08E-01
Actin cytoskeleton (GO:0015629)	77	41	19.27	+	2.13	1.81E-04	8.61E-04
Lysosome (GO:0005764)	24	12	6.01	+	2.00	5.84E-02	1.57E-01
Vesicle coat (GO:0030120)	17	8	4.25	+	1.88	1.37E-01	2.92E-01
Nuclear envelope (GO:0005635)	55	22	13.76	+	1.60	6.41E-02	1.66E-01
Ribonucleoprotein complex (GO:0030529)	348	132	87.09	+	1.52	6.21E-05	3.85E-04
Cytoskeleton (GO:0005856)	263	100	65.82	+	1.52	5.38E-04	2.22E-03
Extracellular matrix (GO:0031012)	29	11	7.26	+	1.52	2.37E-01	4.07E-01
Microtubule organizing center (GO:0005815)	48	18	12.01	+	1.50	1.64E-01	3.17E-01
Ribosome (GO:0005840)	139	52	34.79	+	1.49	1.76E-02	5.47E-02
Nuclear chromosome (GO:0000228)	72	25	18.02	+	1.39	1.62E-01	3.23E-01
Endosome (GO:0005768)	55	19	13.76	+	1.38	2.43E-01	4.07E-01
Nucleoplasm (GO:0005654)	245	84	61.32	+	1.37	1.47E-02	4.79E-02
Peroxisome (GO:0005777)	41	14	10.26	+	1.36	3.12E-01	4.50E-01
Microtubule (GO:0005874)	77	26	19.27	+	1.35	2.16E-01	3.93E-01
Cytoplasmic membrane-bounded vesicle (GO:0016023)	79	26	19.77	+	1.32	2.22E-01	3.93E-01
Endoplasmic reticulum (GO:0005783)	251	81	62.82	+	1.29	5.21E-02	1.47E-01
Vacuole (GO:0005773)	130	42	32.54	+	1.29	1.51E-01	3.12E-01
Golgi apparatus (GO:0005794)	190	61	47.55	+	1.28	9.49E-02	2.18E-01
Mitochondrion (GO:0005739)	270	84	67.57	+	1.24	8.10E-02	2.01E-01

As expected, over 200 localized mRNAs encode proteins destined for the plasma membrane. Many of these mRNAs encode secreted proteins or proteins that are integral to membranes that are trafficked for translation into the ER. However, the localization patterns of these mRNAs included both “basal” and “apical” as well as “cytoplasmic foci,” suggesting that there are different modes of regulation. Mirroring the known roles for mRNA localization in regulating junctional proteins, another overrepresented superset of terms encompassed cytoskeletal elements including “apical cytoskeleton” and “junction.” Of particular interest was that a significantly overrepresented cluster were proteins trafficked to specific organelles including the mitochondria, lysosome, centrosomes/spindles, and peroxisomes.

The role of localized translation at organelles is an emerging area of interest in other organisms and is likely similarly conserved in most cells, including later development. The concept of nucleic acids being targeted to specific organelles to direct protein translation has been suggested in several organisms (reviewed in Weis et al., 2013). Except for mRNAs that encode secreted proteins and are directed to the ER, few functional examples of mRNAs targeted to specific organelles are known in *Drosophila*. Remarkably, the GO-enrichment analysis of mRNAs localized during later *Drosophila* development indicated the prevalence of two terms not previously strongly associated with mRNA localization events: peroxisomes and centrosomes. The association of localized mRNAs cells with centrosomes is particularly interesting in light of how mRNA localization contributes to defining spindle orientation and differentiation of neuronal stem cells, as described above. However, in some cases the observed localization pattern of the mRNA correlates directly with the organelle, suggesting local regulation of translation. In other cases, the lack of correlation of the mRNA location with the organelle, suggests other regulatory events. Below we consider three examples of potential roles for mRNA localization in regulating cell cortex/junctional mediated polarity, peroxisome or centrosomes during later *Drosophila* development.

## Many Localized mRNAs Encode Cell Cortex and Junction Proteins

Given the previous demonstrations of a functional role for transcript localization for the junctional proteins *baz, std*, and *crb*, it is perhaps not particularly surprising that the mRNAs encoding other proteins involved in the establishment or maintenance of cellular junctions are also localized. The mRNA encoding Moe is concentrated apically in follicle cells at stage 10 (Jambor et al., 2015). Moe is the single fly ortholog of Ezrin-Radixin-Moesin (ERM) proteins that link the apical membrane to the cortical actin cytoskeleton (Solinet et al., 2013). There is some evidence that Moe may also regulate mRNA export from the nucleus (Kristó et al., 2017). More notably, Moe interacts directly with other proteins encoded by mRNAs localized apically in cellularized embryos, including Crb and Patj (Médina et al., 2002). The Moe protein has also been reported to interact with Eb1, Chic (profillin) (Medioni et al., 2014), and Chd64 a transgelin 2 ortholog (Guruharsha et al., 2011). The mRNAs encoding each of these proteins are localized apically in follicle cells (Jambor et al., 2015). Notably, *Chd64* mRNA shows basal enrichment in stage 6, 7 embryos (Wilk et al., 2016), implying a potential regulatory mechanism sequestering translation away from the apical membrane. The *Patj* mRNA is localized adjacent to the apical cell junction in the epithelial cells in stage 4–17 embryos (Wilk et al., 2016). Patj interacts with Par-6, Sdt, and aPKC, again all encoded by mRNAs localized apically. Similarly, the *expanded* (*ex*) mRNA is localized apically in stage 2–10 follicle cells (Jambor et al., 2015). Ex is an EPB41/Protein 4.1+ERM (FERM) domain protein that localizes to apical cell-cell junctions (McCartney et al., 2000). Merlin (Mer) is an apically localized regulator of the adherens junction (Lajeunesse et al., 1998) and the *Mer* mRNA is localized apically in follicle cells (Jambor et al., 2015). The mRNA encoding Dlg1 described earlier is also localized at the membrane in late-stage embryos (Wilk et al., 2016).

Many mRNAs that have roles in embryo epithelial cell or follicle cell polarity including the organization of the cellular cortex are localized in *Drosophila*. The mRNA encoding *Drosophila* Shroom is localized apically in stage 2–8 follicle cells and cellularized embryos (stage 6–9) (Jambor et al., 2015; Wilk et al., 2016). *Shroom* encodes two isoforms, both proteins localized apically, one to the adherens junction and one to apical membrane (Bolinger et al., 2010). Shroom proteins regulate cell morphology in animals, by acting on the actin/myosin network during gastrulation (Lee et al., 2009). Similarly, the *Dystrophin* (*Dys*) mRNA is restricted to the cortex of embryo epithelia, and similar cortical enrichment is seen in follicle cells, somatic cells and border cells (stage 9–10) (Jambor et al., 2015; Wilk et al., 2016). Dys is best known for its role in anchoring membrane/cytoskeletal elements in contractile muscle (Constantin, 2014). However, *Drosophila* Dys is also involved in establishing cellular polarity in imaginal discs and oocytes (Dekkers et al., 2004). Notably, a similar role for Dystrophin has also been shown for mammalian muscle stem cells (Keefe and Kardon, 2015). Another mRNA with cortical localization in the embryo is *Tropomyosin 1* (*Tm1*), encoding a protein involved in muscle contraction, oogenesis, and regulation of *osk* mRNA localization (Erdélyi et al., 1995; Veeranan-Karmegam et al., 2016; Gáspár et al., 2017). Several other actin-interacting proteins have mRNAs with cortical localization in follicle cells including Jitterbug (Jbug, *Drosophila* filamin) (Jambor et al., 2015). However, unlike what has been shown previously, co-localization of mRNAs encoding these junctional proteins are not facilitating local protein translation. In the case of the *Jbug* and *short stop* (*shot*) encoding *Drosophila* Spectraplakin, both mRNAs localized basally in Stage 6–9 embryo ectodermal cells (Wilk et al., 2016), away from the region where the protein is localized. Other apically localized mRNAs that encode proteins that interact with cytoskeletal elements include Scraps (Scra, orthologous to Anillin), the *Drosophila* ortholog of Facin Actin-bundling protein 1 Singed (Sn) and α-actinin (Actn) (Jambor et al., 2015; Wilk et al., 2016).

## Peroxisomes

Peroxisomes are cytosolic organelles involved in lipid metabolism and detoxifying reactive oxygen species (Smith and Aitchison, 2013). The *Peroxin* (*Pex*) genes encode proteins involved in peroxisome biogenesis, a process that includes vesicles budding the ER that fuse and mature by importing peroxisome enzymes (*Pex1* and *Pex5*) or peroxisome fission (*Pex11*) and are conserved in *Drosophila* (Baron et al., 2016). It has been proposed that localization of *Pex* mRNAs to peroxisomes may direct translation into the peroxisomal membrane tethering them in a fashion reminiscent of ER-protein targeting (Haimovich et al., 2016a). Pex proteins are highly conserved between yeast; humans and *Drosophila* (Mast et al., 2011; Faust et al., 2014; Baron et al., 2016). Traditional FISH screens identified multiple mRNAs encoding peroxisome-associated proteins as localized in various cell lineages in late-stage *Drosophila* embryos (Table 2). However, correlating a functional role between the location of mRNAs and their product is difficult as encoded proteins involved in the same process are trafficked to different cytoplasmic regions (e.g., Pex16 and Pex19). Particularly interesting is the pattern of *Pex5* mRNA encoding the cytoplasmic transporter that directs proteins to the peroxisome. *Pex5* mRNA as it was observed in foci, surrounding the nucleus while the mRNA encoding the Pex5 recycling protein Pex1 is restricted apically. In addition to the Peroxins, there are also 20 localized mRNAs encoding peroxisome resident enzymes including *Catalase* (*Cat*, Table 2) (Wilk et al., 2016).

**Table 2:** Localized mRNAs encoding peroxisome proteins Wilk et al. (2013).

**Gene**		**Localization**	**Stage**
**PEROXISOMES**			
***Peroxins***			
*Pex1*	*Peroxin1*	Basal	Stage 4-7
*Pex3*	*Peroxin3*	Cortex	Stage 6-7
*Pex5*	*Peroxin5*	Basal	Stage 4-7
*Pex11*	*Peroxin11*	Basal	Stage 4-7
*Pex14*	*Peroxin14*	Medial	Stage 4-7
*Pex16*	*Peroxin16*	Perinuclear foci	Larval fat body, malpighian tubules, testes
*Pex19*	*Peroxin19*	Apical	Stage 4-7
***Peroxisomal Enzymes***			
*Acox57D-d*	*acyl-Coenzyme A oxidase at 57D distal*	Basal/perinuclear	Stage 8-9
*Acsl*	*Acyl-CoA synthetase long-chain*	Basal/cytoplasmic foci	Stage 4-17
*Best2*	*Bestrophin 2*	Cytoplasmic foci	
*Cat*	*Catalase*	Apical	Stage 10-11
*CG10096*	*FAR1 like*	Basal	Stage 4-17
*CG10932*	*ACAT1*	Cytoplasmic foci	Stage 4-9
*CG12338*	*Dao*	Apical exclusion	Stage 4-5
*CG12428*	*Crot*	Apical exclusion	Stage 4-5
*CG13890*	*Pec1*	Perinuclear foci	Malpighian tubules
		Medial	Stage 4-5
*CG31259*	*TMEM135*	Apical exclusion	Stage 4-5
*CG5009*	*ACOX1*	Apical exclusion / nuclei	Stage 4-8
*CG9149*	*ACAA1*	Apical exclusion	Stage 4-5
*CG9577*	*ECH1*	Basal / membrane associated	Stage 6-9
*Got1*	*Glutamate oxaloacetate transaminase 1*	Basal	Stage 6-7
		Cytoplasmic foci	Larval fat body, malpighian tubules
*Idh*	*Isocitrate dehydrogenase*	Cytoplasmic foci	Stage 4-7, larval fat body, malpighian tubules
*Lon*	*Lon protease*	Basal	Stage 8-9
*Mdh1*	*Malate dehydrogenase 1*	Basal	Stage 4-17, midgut, membrane associated
		Apical	Stage 10-17 hindgut
*Mtpα*	*Mitochondrial trifunctional protein α subunit*	Perinuclear foci	Stage 6-17
*Mul1*	*Mitochondrial E3 ubiquitin protein ligase 1*	Apical exclusion	Stage 4-5
*Pi3K59F*	*Phosphatidylinositol 3 kinase 59F*	Basal	Stage 6-7
*Sod1*	*Superoxide dismutase 1*	Apical exclusion	Stage 4-7
*Wat*	*Waterproof*	Basal	Stage 6-7
		Cytoplasmic foci	Stage 8-9

Studies in yeast have also shown that a significant number of *peroxin* mRNAs localize to peroxisomes or other peroxisome-associated organelles (e.g., Pex3 and the ER). In yeast, the Puf5 RNA binding protein, related to *Drosophila* Pum is required for Pex14 localization to the peroxisome (Zipor et al., 2009). Puf5p also binds the yeast-specific *Pex22* mRNA (Gerber et al., 2004). It has been proposed that the association of mRNAs encoding cytoplasmic Pex proteins with peroxisomes foster local translation and Insertion incorporation of peroxisomal membrane proteins (Weis et al., 2013; Haimovich et al., 2016a). However, other mRNAs associated with the exterior membrane of peroxisomes isolated from mouse liver were also identified (Yarmishyn et al., 2016). This included mRNAs encoding *Pex6, Pex11a*/b, and *Pex19*, and *Peroxisomal Membrane Protein 70 kDa (Pmp 70)* as well as mRNAs encoding homologs to several peroxisomal localized enzymes including *Hmgcs1, Acaa1a*/*b, Hsd14b4, Paox, Nudt7, Acox, Baat*, and *Acsl5* as peroxisome associated (Yarmishyn et al., 2016). However, FISH was used to confirm peroxisome localization of only one of these mRNAs, *Hmgcs1* (Yarmishyn et al., 2016). While the prevalence of mRNA localization of peroxisome mRNAs is striking and suggests a functional role, the localization pattern of these mRNAs encompasses apical and basal restriction, perinuclear patterns and cytoplasmic foci in embryo ectoderm and various larval tissues (Wilk et al., 2016). The conservation of the phenomenon of *Pex* mRNA localization in *Drosophila* provides support that this event may have functional consequences during peroxisome biogenesis, fission or steady state homeostasis.

## Centrosomes

As described above, there are several known roles for mRNA localization, to define the orientation of the mitotic spindle in *Drosophila* neural stem cells. However, systematic FISH assays suggest that mRNAs encoding several components of the centrosome or spindle are themselves localized, suggesting a more direct role in mRNA localization. Centrosomes are found exclusively in metazoan cells (Bornens, 2012). Centrosomes encapsulate centrioles in an electron-dense pericentriolar material (PCM) of dynamic composition and size (Brito et al., 2012). During interphase, the centrosome acts as the primary cellular microtubule-organizing center (MTOC) involved in cellular trafficking, motility, adhesion, and polarity, while during mitosis, they help establish the spindle. Following mitosis, the centrosome contains both a mature centriole (mother) and a newly formed immature centriole assembled during the previous cell cycle (daughter). Assembly of the daughter centrosome occurs during S-phase, through recruitment of PCM proteins to the daughter centrioles (Gogendeau and Basto, 2010; Nigg and Stearns, 2011; Brito et al., 2012; Habermann and Lange, 2012; Mahen and Venkitaraman, 2012).

A functional role for recruitment of mRNAs to the centrosome/spindle has been posited and then subsequently discounted several times. The first general reports of nucleic acids within the centrosome were made in the mid-twentienth century (Stich, 1954; Ota and Shimamura, 1956; Rustad, 1959; Zimmerman, 1960; Ackerman, 1961; Hartman et al., 1974; Dippell, 1976; Moyne and Garrido, 1976; Zackroff et al., 1976; Rieder, 1979; Snyder, 1980). RNA as a potential spindle or centrosome component was first described 40 years ago (Heidemann et al., 1977; Peterson and Berns, 1978). However, lacking functional data, these findings were discounted as contamination. The most compelling empirical support for a functional role for mRNA localized at the spindle comes from *Ilyanassa* (snail) embryos (Lambert and Nagy, 2002; Kingsley et al., 2007). Similarly, localization of mRNAs encoded by centrosome genes was observed in *Spisula solidissima* (clam) oocytes (Alliegro et al., 2006, 2010; Alliegro and Alliegro, 2008). Finally, cytoplasmic mRNA regulation is required for normal spindle pole body function in yeast, although it is not known if this is required generally or locally at the MTOC (Unger, 1977; Volpe et al., 2003; Sezen et al., 2009).

Seventy-one *Drosophila* mRNAs localized to the spindle/centrosome in late stage-embryos or follicle cells encoding centrosome, spindle, or centriole associated proteins. Notably, some were annotated (Table 3) with a centrosome or spindle localization pattern (e.g., *Centrocortin, Cen* and *Girdin*), or a perinuclear pattern which would encompass centrosomes (α*-Tubulin at 84B*, α*-Tub84B, pavarotti, pav, centrosomin, Grip91, spindle defective 2, spd2*, and γ*-Tubulin at 37C*). However, some of these mRNAs (α*-Tub84B, Cen, Girdin, pav, spd-2*) as well as others, localized to cytoplasmic foci (*Gamma tubulin ring protein 91, Kinesin-like protein at 10A, non-claret disjunctional, Spindle assembly abnormal 6*, and *scrambled*). The annotation of cytoplasmic foci could encompass centrosomes but could equally encompass other destinations including regulatory RNPs. Notably, the stages or tissues where these were annotated varies considerably (Table 3), implying the potential for developmental regulation as well. The *Drosophila* centrosome proteome is well-characterized facilitating direct correlation of localized mRNAs to centrosomally localized proteins. Müller et al. identified 24 known and 227 previously unknown centrosome-associated proteins via mass spectrometry (Muller et al., 2010). Notably, the mRNAs encoding Aurora A and Polo kinases, involved in regulating spindle formation/mitosis, were localized to cytoplasmic foci and for Polo in a perinuclear pattern (Jambor et al., 2015; Wilk et al., 2016). With the high degree of correlation between centrosome proteins and centrosome/perinuclear localized mRNAs, a role for local regulation of translation for functions related to centrosomes in *Drosophila* is an attractive hypothesis.

**Table 3:** Localized mRNAs encoding centrosome proteins (Wilk et al., 2013).

**Gene**		**Role**	**Localization**	**Stage**
**CENTROSOMES/SPINDLE**				
*αTub84B*	*α-Tubulin at 84B*	Tubulin	Apical localization	Stage 6-17
			Membrane associated	Stage 4-17, imaginal discs
			Perinuclear	Stage 10-17 (ectoderm, trachea)
			Cytoplasmic foci	fat body, gut, proventriculus, lymph glands, muscles, imaginal discs CNS
*aurA*	*aurora A*	Kinase	Cytoplasmic foci	Stage 6-7
*Cen*	*Centrocortin*	Centrosome	Centrosome	Stage 4-9
			Cytoplasmic foci	Stage 10-17, dorsal trunk, brain, salivary gland, CNS, trachea, fat body, imaginal discs, intestine
*cnn*	*centrosomin*	Centrosome	Perinuclear (yolk nuclei)	Stage 4-7
*Girdin*	*Girdin*		Apical clusters	Stage 4-7
			Centrosome	Stage 4-5
			Cytoplasmic foci	Stage 6-9, stage 10 (ventral nerve cord, fat body, brain, midgut, pharynx)
*Grip163*	*Grip163*	Microtubule binding	Cytoplasmic foci	Stage 6-7
*Grip91*	*Gamma-tubulin ring protein 91*	Microtubule binding	Perinuclear	Stage 4-7
*Klp10A*	*Kinesin-like protein at 10A*	Kinesin	Apical exclusion	Stage 6-7
			Cytoplasmic foci (basal)	Stage 8-9
*ncd*	*Non-claret disjunctional*	Kinesin	Cytoplasmic foci	Stage 10-17
*pav*	*Pavarotti*	Kinesin	Cytoplasmic foci (basal)	Stage 6-7
			Perinuclear foci (CNS nuclei)	Stage 10-17
*Polo*	*Polo*	Kinase	Perinuclear (CNS nuclei)	Stage 10-17
			Few cytoplasmic foci	Stage 10-17 (brain), fat body, imaginal discs, hindgut, midgut, muscles, malpighian tubules
*Sas-6*	*Spindle assembly abnormal 6*	Centriole	Cytoplasmic foci	Stage 6-9
			Foci in ectoderm	Stage 10-17
*sced*	*Scrambled*	Pseudocleavage furrow	Cytoplasmic foci	Stage 8-9
*spd-2*	*Spindle defective 2*	Centriole/centrosome	Cytoplasmic foci	Stage 10-17, (brain), tracheal, dorsal trunk, head
			Perinuclear	Stage 4-5, 10-17
*γTub37C*	*γ-Tubulin at 37C*	Centrosome localized tubulin	Perinuclear (CNS nuclei)	Stage 10-17

### Looking to the Future: *Drosophila* Is Well-Positioned to Advance Understanding of the Role of mRNA Localization During Later Development

The *Drosophila* oocyte and early embryo provided a wealth of knowledge regarding the prevalence, regulation and functional roles for localized mRNAs during early development. What remains to be determined is if this regulatory event is similarly functionally prevalent during later development. It is known that localized mRNA have regulates polarized neural lineages. It will be interesting to see what roles localized mRNAs have in other stem-cell populations and the various other polarized cell lineages required for development into the adult form. The examples considered above are only a sample of the thousands of mRNAs annotated as localized in one or more cell types during later stage *Drosophila* development (Figure 1), yet they are indicative of the existence of yet-to-be-discovered regulatory examples. The current challenge in the field is to now to determine which of the long list of transcripts that show sub-cellular localization, represents functional regulatory events.

Unfortunately, in most cases, the phenomenon of localization has not been linked to the effect of mRNA translation or degradation. It is possible to assay the translation state of a specific mRNA by determining the presence or absence of ribosomes. Traditionally, the stage of translation has been tested by profiling the mRNAs associated with purified polysomes (reviewed in Chassé et al., 2017; Seimetz et al., 2018), but this runs the risk of not detecting specific local differences in translation. Recently, several new methods have been reported that link single molecule RNA (smRNA) imaging to detection of ribosomes on that specific mRNA. The first of these, “translating RNA imaging by coat protein knockoff” (TRICK) was shown to be viable in *Drosophila* oocytes (Halstead et al., 2015). TRICK detects the initial passage of ribosomes along the ORF of an mRNA expressed specially constructed reporter. These mRNAs can be individually tracked in live cells by 24x MS2 aptamers in the 3′UTR. The ribosome passage will displace fluorescent protein reporters associated with 6xPP7 aptamer sequences cloned in frame to the protein sequence. The fluorescent PP7 binding protein was displaced by the passage of ribosome along the ORF of the reporter. In 2016, several groups published a combination of methods combining mRNA aptamer-based detection of single mRNAs in living cells with different methods to detect ongoing protein translation (Morisaki et al., 2016; Pichon et al., 2016; Wang et al., 2016; Wu et al., 2016; Yan et al., 2016). However, the utility of these methods to study gastrulating embryos or dissected larval tissues has not yet been established as they all rely on single-molecule imaging using microscope techniques developed for relatively thin tissues or individual cells. These aptamer-tagging methods should be approached with caution, however. Recently, several groups have suggested caution in interpreting the localization or degradation of mRNAs including these tags. There has been a considerable back-and-forth regarding the consequences of introducing aptamer tags that would recruit a large protein complex (e.g., multiple MS2-GFP) affecting mRNA stability and localization (Garcia and Parker, 2015, 2016; Haimovich et al., 2016b; Heinrich et al., 2017b). Recently, the Singer laboratory has developed a modified form of the MS2 aptamer that should allow for more “normal” recruitment to mRNA processing bodies or mRNA localization (Tutucci et al., 2018). These live-cell methods to track mRNAs can be coupled to complementary methods that image newly synthesized proteins include fluorescent non-canonical amino acid tagging (FUNCAT) using a modified methionine analog (Tom Dieck et al., 2015) or tetracysteine (TC) motifs that bind biarsenical fluorescent dyes (Rodriguez et al., 2006).

Alternative approaches to tracking individual mRNAs in live cells employ aptamers that bind and induce fluorescence of various chemicals (e.g., Broccoli or RNA Mango) have been developed. These would not suffer from the effect of recruiting additional protein complexes to an mRNA (Paige et al., 2011; Dolgosheina et al., 2014; Filonov et al., 2014; Autour et al., 2018). One of the newest of these chemical/aptamer systems that shows promise is Riboglow, a riboswitch based system that recruits a Cobalamin + fluorophore combination (Braselmann et al., 2018). Cobalamin effectively quenches the fluorophore until bound by the RNA target. While Riboglow has shown promise with respect to signal-to-noise, it requires bead loading to get the detection reagent into cells, which may limit use in *Drosophila* late-stage embryos and larval tissues. Overall, the potential advantage of chemical/aptamer systems over aptamer/protein-FP combinations is that a chemical/aptamer complex is significantly smaller than a fluorescent protein/aptamer complex. A second advantage is that with chemical/aptamer pairs, fluorescence is only induced upon target binding. The major disadvantage is that unlike MS2 or similar systems, the fluorescent signal, especially using single aptamers is relatively weak. Thus, these work well for relatively highly expressed RNAs like rRNAs but have not yet been shown to be practical for relatively less common mRNAs, nor would they be sufficient for imaging mRNA localization within complex tissues. Similar to aptamer-based live-cell mRNA detection; these methods can also be coupled with complementary detection of newly translated protein.

All of these methods described above, correlating translation to a localized mRNA, depend on expressing transgenes that express highly modified mRNAs with multiple different inserted motifs, making them relatively impractical for high-throughput approaches. However, a method to correlate ribosomes and mRNAs expressed from endogenous, unmodified genes in fixed cells based on smFISH to ribosome RNAs, has recently been described that would be practical for high-throughput use in whole fly embryos analogous to the FISH screens described above. The FLorescent Assay to detect Ribosome Interactions with mRNA (FLARIM) (Burke et al., 2017) is an extension of the single molecule hybridization chain reaction (HCR), (Shah et al., 2016). This method uses two different smFISH probe sets detecting the ORF and the 18S rRNA marking the ribosome (Burke et al., 2017). FLARIM uses smHCR for smRNA detection of the target and 18S RNA detection, but other smFISH such as quantitative Forced InTeraction (qFIT) or other low-cost FISH-based procedures should also be similarly effective (Gaspar et al., 2017). Detection of localized mRNA decay is also possible, via co-localization of specific transcripts with known cytoplasmic RNPs, (reviewed in Towler and Newbury, 2018). Additionally, smFISH approaches have also been shown to be able to measure mRNA decay in yeast or trypanosomes (Kramer, 2017; Trcek et al., 2018), although these have not yet been adapted to *Drosophila*.

The other recent advance that will facilitate examination of the functional roles for mRNA localization in the relatively smaller cells that comprise the bulk of later embryo, larval and adult development is super resolution (SR) microscopy. These techniques have now been adapted to Drosophila (reviewed in Rodal et al., 2015) Recently, 3D-Structured Illumination Microscopy (SIM), using the Deltavision OMX system, was used to detect smFISH signals at the Drosophila NMJ (Titlow et al., 2018). Another SR technique that show promise for live imaging in later stage *Drosophila* embryos is lattice light-sheet imaging (LLSM) (Planchon et al., 2011; Chen et al., 2014). Other LSM methods that have recently been shown to be suitable for imaging later *Drosophila* embryos (e.g., +15 h) include reflected LSM (R-LSM) (Greiss et al., 2016) and Tilted LSM (TLSM) (Fadero et al., 2018; Gustavsson et al., 2018). An alternative method to improve resolution within cells of later stage *Drosophila* embryos or tissues is expansion microscopy (ExM). ExM effectively makes cells/tissues larger via treatment with polymer hydrogels (Chen et al., 2015) ExM has been shown to be compatible with smFISH methods including HCR FISH, facilitating imaging mRNA localization in cells deep within tissues (Chen et al., 2016). ExM has recently been shown to be feasible for examining later stages of fly development including such tissues as the adult brain (Mosca et al., 2017; Gao et al., 2019). Most importantly for the study of late stage fly development, ExM can be performed in tandem with enzymatic digestion of the embryo cuticle, facilitating tissue expansion in late-stage embryos (Jiang et al., 2018).

### Identifying the Mechanisms That Localize mRNAs During Later Development

While outside the scope of this review, the other major challenge that remains is to determine the conservation of mechanisms that direct mRNA to specific cytoplasmic regions in the various cells of the late-stage embryo, larva or adult and what roles this localization plays in regulating the encoded protein. Since the FlyBase release FB2018_03 (June 16, 2018), there are 913 known RNA binding proteins encoded by the *Drosophila* genome (Gramates et al., 2017). Many of the proteins known to localize during oocyte or early embryo development are expressed in one or more lineages in later development. Notably, there have been several advances in identifying protein binding to specific RNAs that would be compatible for use in complex tissues (Lee et al., 2013; Di Tomasso et al., 2016; Autour et al., 2018), reviewed in (Faoro and Ataide, 2014).

### The Future Is Bright for Studying mRNA Localization During Later *Drosophila* Development

The study of mRNA localization in *Drosophila*, especially during later development, is at an exciting crossroad. The wealth of data from traditional FISH-based screens provides a valuable resource outlining the full scope of localized mRNAs, encoding proteins involved in multiple cellular processes, and the possibility that these processes may be linked to localized transcripts in other organisms. The advent of contemporary smFISH techniques, including those that can also locally detect translational states, provide viable avenues to correlate existing phenomenological observation to the functional roles. Recent improvements in both microscope resolution available for fluorescent imaging, as well as the advent of workflows for robust and practical three-dimensional electron-microscope imaging (Xu et al., 2017), will improve the capacity to observe sub-cellular restriction mRNA restriction and specific organelle localization, especially in the relatively smaller-sized epithelial cell lineages that form developing tissues. These methods will help link localization of mRNAs during later stage *Drosophila* development to what is undoubtedly an equally broad spectrum of functional consequences of the proteins they encode.

## Author Contributions

All authors listed have made a substantial, direct and intellectual contribution to the work, and approved it for publication.

### Conflict of Interest Statement

The authors declare that the research was conducted in the absence of any commercial or financial relationships that could be construed as a potential conflict of interest.

## References

[B1] Abaza I. Gebauer F. (2008). Trading translation with RNA-binding proteins. RNA 14, 404–409. 10.1261/rna.848208 18212021 PMC2248257

[B2] Abbaszadeh E. K. Gavis E. R. (2016). Fixed and live visualization of RNAs in *Drosophila* oocytes and embryos. Methods 98, 34–41. 10.1016/j.ymeth.2016.01.018 26827935 PMC4808400

[B3] Abeysundara N. Simmonds A. J. Hughes S. C. (2018). Moesin is involved in polarity maintenance and cortical remodeling during asymmetric cell division. Mol. Biol. Cell 29, 419–434. 10.1091/mbc.E17-05-0294 29282284 PMC6014166

[B4] Ackerman G. A. (1961). Histochemistry of the centrioles and centrosomes of the leukemic cells from human myeloblastic leukemia. J. Biophys. Biochem. Cytol. 11, 717–719. 10.1083/jcb.11.3.717 13859170 PMC2225139

[B5] Agnès F. Perron M. (2004). RNA-binding proteins and neural development: a matter of targets and complexes. Neuroreport 15, 2567–2570. 10.1097/00001756-200412030-00001 15570153

[B6] Alliegro M. A. Henry J. J. Alliegro M. C. (2010). Rediscovery of the nucleolinus, a dynamic RNA-rich organelle associated with the nucleolus, spindle, and centrosomes. Proc. Natl. Acad. Sci. U.S.A. 107, 13718–13723. 10.1073/pnas.1008469107 20643950 PMC2922224

[B7] Alliegro M. C. Alliegro M. A. (2008). Centrosomal RNA correlates with intron-poor nuclear genes in Spisula oocytes. Proc. Natl. Acad. Sci. U.S.A. 105, 6993–6997. 10.1073/pnas.0802293105 18458332 PMC2383940

[B8] Alliegro M. C. Alliegro M. A. Palazzo R. E. (2006). Centrosome-associated RNA in surf clam oocytes. Proc. Natl. Acad. Sci. U.S.A. 103, 9034–9038. 10.1073/pnas.0602859103 16754862 PMC1482561

[B9] Asaoka-Taguchi M. Yamada M. Nakamura A. Hanyu K. Kobayashi S. (1999). Maternal pumilio acts together with Nanos in germline development in *Drosophila* embryos. Nat. Cell Biol. 1, 431–437. 10.1038/15666 10559987

[B10] Ashburner M. Ball C. A. Blake J. A. Botstein D. Butler H. Cherry J. M. (2000). Gene ontology: tool for the unification of biology. The gene ontology consortium. Nat. Genet. 25, 25–29. 10.1038/75556 PMC3037419 10802651

[B11] Autour A. C. Y. Jeng S. Cawte A. D. Abdolahzadeh A. Galli A. Panchapakesan S. S. S.. (2018). Fluorogenic RNA Mango aptamers for imaging small non-coding RNAs in mammalian cells. Nat. Commun. 9:656. 10.1038/s41467-018-02993-8 29440634 PMC5811451

[B12] Bachmann A. Schneider M. Theilenberg E. Grawe F. Knust E. (2001). *Drosophila* stardust is a partner of crumbs in the control of epithelial cell polarity. Nature 414, 638–643. 10.1038/414638a 11740560

[B13] Baron M. N. Klinger C. M. Rachubinski R. A. Simmonds A. J. (2016). A systematic cell-based analysis of localization of predicted *Drosophila* peroxisomal proteins. Traffic 17, 536–553. 10.1111/tra.12384 26865094

[B14] Bayer L. V. Batish M. Formel S. K. Bratu D. P. (2015). Single-molecule RNA *in situ* hybridization (smFISH) and immunofluorescence (IF) in the *Drosophila* egg chamber. Methods Mol. Biol. 1328, 125–136. 10.1007/978-1-4939-2851-4_9 26324434

[B15] Bergeman J. Caillier A. Houle F. Gagné L. M. Huot M.-É. (2016). Localized translation regulates cell adhesion and transendothelial migration. J. Cell Sci. 129, 4105. 10.1242/jcs.191320 27637266

[B16] Bertrand E. Chartrand P. Schaefer M. Shenoy S. M. Singer R. H. Long R. M. (1998). Localization of *ASH1* mRNA particles in living yeast. Mol. Cell 2, 437–445. 10.1016/S1097-2765(00)80143-4 9809065

[B17] Bhat M. A. Izaddoost S. Lu Y. Cho K. O. Choi K. W. Bellen H. J. (1999). Discs lost, a novel multi-PDZ domain protein, establishes and maintains epithelial polarity. Cell 96, 833–845. 10.1016/S0092-8674(00)80593-0 10102271

[B18] Bolinger C. Zasadil L. Rizaldy R. Hildebrand J. D. (2010). Specific isoforms of *Drosophila* shroom define spatial requirements for the induction of apical constriction. Dev. Dyn. 239, 2078–2093. 10.1002/dvdy.22326 20549743 PMC2913297

[B19] Bornens M. (2012). The centrosome in cells and organisms. Science 335, 422–426. 10.1126/science.1209037 22282802

[B20] Braselmann E. Wierzba A. J. Polaski J. T. Chrominski M. Holmes Z. E. Hung S. T.. (2018). A multicolor riboswitch-based platform for imaging of RNA in live mammalian cells. Nat. Chem. Biol. 14, 964–971. 10.1038/s41589-018-0103-7 30061719 PMC6143402

[B21] Brechbiel J. L. Gavis E. R. (2008). Spatial regulation of *nanos* is required for its function in dendrite morphogenesis. Curr. Biol. 18, 745–750. 10.1016/j.cub.2008.04.033 18472422 PMC2474551

[B22] Brennan M. D. Weiner A. J. Goralski T. J. Mahowald A. P. (1982). The follicle cells are a major site of vitellogenin synthesis in *Drosophila melanogaster*. Dev. Biol. 89, 225–236. 10.1016/0012-1606(82)90309-8 6172303

[B23] Brito D. A. Gouveia S. M. Bettencourt-Dias M. (2012). Deconstructing the centriole: structure and number control. Curr. Opin. Cell Biol. 24, 4–13. 10.1016/j.ceb.2012.01.003 22321829

[B24] Broadus J. Fuerstenberg S. Doe C. Q. (1998). Staufen-dependent localization of prospero mRNA contributes to neuroblast daughter-cell fate. Nature 391, 792–795. 10.1038/35861 9486649

[B25] Brown J. B. Celniker S. E. (2015). Lessons from modENCODE. Annu. Rev. Genomics Hum. Genet. 16, 31–53. 10.1146/annurev-genom-090413-025448 26133010

[B26] Buchan J. R. Parker R. (2009). Eukaryotic stress granules: the ins and outs of translation. Mol. Cell 36, 932–941. 10.1016/j.molcel.2009.11.020 20064460 PMC2813218

[B27] Burke K. S. Antilla K. A. Tirrell D. A. (2017). A Fluorescence *in situ* hybridization method to quantify mRNA translation by visualizing ribosome–mRNA interactions in single cells. ACS Central Sci. 3, 425–433. 10.1021/acscentsci.7b00048 28573204 PMC5445550

[B28] Cavey M. Lecuit T. (2009). Molecular bases of cell-cell junctions stability and dynamics. Cold Spring Harb. Perspect. Biol. 1:a002998. 10.1101/cshperspect.a002998 20066121 PMC2773640

[B29] Chandra S. Vimal D. Sharma D. Rai V. Gupta S. C. Chowdhuri D. K. (2017). Role of miRNAs in development and disease: lessons learnt from small organisms. Life Sci. 185, 8–14. 10.1016/j.lfs.2017.07.017 28728902

[B30] Chassé H. Boulben S. Costache V. Cormier P. Morales J. (2017). Analysis of translation using polysome profiling. Nucleic Acids Res. 45:e15. 10.1093/nar/gkw907 28180329 PMC5388431

[B31] Chen B. C. Legant W. R. Wang K. Shao L. Milkie D. E. Davidson M. W.. (2014). Lattice light-sheet microscopy: imaging molecules to embryos at high spatiotemporal resolution. Science 346:1257998. 10.1126/science.1257998 25342811 PMC4336192

[B32] Chen F. Tillberg P. W. Boyden E. S. (2015). Expansion microscopy. Science 347, 543–548. 10.1126/science.1260088 25592419 PMC4312537

[B33] Chen F. Wassie A. T. Cote A. J. Sinha A. Alon S. Asano S.. (2016). Nanoscale imaging of RNA with expansion microscopy. Nat. Methods 13, 679–684. 10.1038/nmeth.3899 27376770 PMC4965288

[B34] Cho P. F. Gamberi C. Cho-Park Y. A. Cho-Park I. B. Lasko P. Sonenberg N. (2006). Cap-dependent translational inhibition establishes two opposing morphogen gradients in *Drosophila* embryos. Curr. Biol. 16, 2035–2041. 10.1016/j.cub.2006.08.093 17055983 PMC2238800

[B35] Consortium G. O. (2017). Expansion of the gene ontology knowledgebase and resources. Nucleic Acids Res. 45, D331–D338. 10.1093/nar/gkw1108 27899567 PMC5210579

[B36] Constantin B. (2014). Dystrophin complex functions as a scaffold for signalling proteins. Biochim. Biophys. Acta 1838, 635–642. 10.1016/j.bbamem.2013.08.023 24021238

[B37] Cui X. A. Palazzo A. F. (2014). Localization of mRNAs to the endoplasmic reticulum. Wiley Interdiscip. Rev. RNA 5, 481–492. 10.1002/wrna.1225 24644132

[B38] Dekkers L. C. Van Der Plas M. C. Van Loenen P. B. Den Dunnen J. T. Van Ommen G. J. Fradkin L. G.. (2004). Embryonic expression patterns of the *Drosophila* dystrophin-associated glycoprotein complex orthologs. Gene Expr. Patterns 4, 153–159. 10.1016/j.modgep.2003.09.004 15161095

[B39] Di Tomasso G. Miller Jenkins L. M. Legault P. (2016). ARiBo pull-down for riboproteomic studies based on label-free quantitative mass spectrometry. RNA 22, 1760–1770. 10.1261/rna.057513.116 27659051 PMC5066628

[B40] Ding D. Parkhurst S. M. Halsell S. R. Lipshitz H. D. (1993). Dynamic Hsp83 RNA localization during *Drosophila* oogenesis and embryogenesis. Mol. Cell. Biol. 13:3773. 10.1128/MCB.13.6.3773 7684502 PMC359859

[B41] Dippell R. V. (1976). Effects of nuclease and protease digestion on the ultrastructure of *Paramecium* basal bodies. J. Cell Biol. 69, 622–637. 10.1083/jcb.69.3.622 178669 PMC2109711

[B42] Dolgosheina E. V. Jeng S. C. Panchapakesan S. S. Cojocaru R. Chen P. S. Wilson P. D.. (2014). RNA mango aptamer-fluorophore: a bright, high-affinity complex for RNA labeling and tracking. ACS Chem. Biol. 9, 2412–2420. 10.1021/cb500499x 25101481

[B43] Dos Santos G. Simmonds A. J. Krause H. M. (2008). A stem-loop structure in the *wingless* transcript defines a consensus motif for apical RNA transport. Development 135, 133–143. 10.1242/dev.014068 18045835

[B44] Doyle M. Kiebler M. A. (2011). Mechanisms of dendritic mRNA transport and its role in synaptic tagging. EMBO J. 30, 3540–3552. 10.1038/emboj.2011.278 21878995 PMC3181491

[B45] Dubowy J. Macdonald P. M. (1998). Localization of mRNAs to the oocyte is common in *Drosophila* ovaries. Mech. Dev. 70, 193–195. 10.1016/S0925-4773(97)00185-8 9510035

[B46] Erdélyi M. Michon A. M. Guichet A. Glotzer J. B. Ephrussi A. (1995). Requirement for *Drosophila* cytoplasmic tropomyosin in *oskar* mRNA localization. Nature 377, 524–527. 10.1038/377524a0 7566149

[B47] Eritano A. S. Altamirano A. Beyeler S. Gaytan N. Velasquez M. Riggs B. (2017). The endoplasmic reticulum is partitioned asymmetrically during mitosis before cell fate selection in proneuronal cells in the early *Drosophila* embryo. Mol. Biol. Cell 28, 1530–1538. 10.1091/mbc.e16-09-0690 28381427 PMC5449151

[B48] Fadero T. C. Gerbich T. M. Rana K. Suzuki A. Disalvo M. Schaefer K. N.. (2018). LITE microscopy: tilted light-sheet excitation of model organisms offers high resolution and low photobleaching. J. Cell Biol. 217, 1869–1882. 10.1083/jcb.201710087 29490939 PMC5940309

[B49] Faoro C. Ataide S. F. (2014). Ribonomic approaches to study the RNA-binding proteome. FEBS Lett. 588, 3649–3664. 10.1016/j.febslet.2014.07.039 25150170

[B50] Faust J. E. Manisundaram A. Ivanova P. T. Milne S. B. Summerville J. B. Brown H. A.. (2014). Peroxisomes are required for lipid metabolism and muscle function in *Drosophila melanogaster*. PLoS ONE 9:e100213. 10.1371/journal.pone.0100213 24945818 PMC4063865

[B51] Femino A. M. Fogarty K. Lifshitz L. M. Carrington W. Singer R. H. (2003). Visualization of single molecules of mRNA *in situ*. Meth. Enzymol. 361, 245–304. 10.1016/S0076-6879(03)61015-3 12624916

[B52] Filonov G. S. Moon J. D. Svensen N. Jaffrey S. R. (2014). Broccoli: rapid selection of an RNA mimic of green fluorescent protein by fluorescence-based selection and directed evolution. J. Am. Chem. Soc. 136, 16299–16308. 10.1021/ja508478x 25337688 PMC4244833

[B53] Gao R. Asano S. M. Upadhyayula S. Pisarev I. Milkie D. E. Liu T.. (2019). Cortical column and whole-brain imaging with molecular contrast and nanoscale resolution. Science 363:eaau8302. 10.1126/science.aau8302 30655415 PMC6481610

[B54] Garcia J. F. Parker R. (2015). MS2 coat proteins bound to yeast mRNAs block 5' to 3' degradation and trap mRNA decay products: implications for the localization of mRNAs by MS2-MCP system. RNA 21, 1393–1395. 10.1261/rna.051797.115 26092944 PMC4509929

[B55] Garcia J. F. Parker R. (2016). Ubiquitous accumulation of 3' mRNA decay fragments in Saccharomyces cerevisiae mRNAs with chromosomally integrated MS2 arrays. RNA 22, 657–659. 10.1261/rna.056325.116 27090788 PMC4836640

[B56] Gáspár I. Sysoev V. Komissarov A. Ephrussi A. (2017). An RNA-binding atypical tropomyosin recruits kinesin-1 dynamically to oskar mRNPs. EMBO J. 36, 319–333. 10.15252/embj.201696038 28028052 PMC5286366

[B57] Gaspar I. Wippich F. Ephrussi A. (2017). Enzymatic production of single-molecule FISH and RNA capture probes. RNA 23, 1582–1591. 10.1261/rna.061184.117 28698239 PMC5602115

[B58] Gavis E. R. Lehmann R. (1992). Localization of nanos RNA controls embryonic polarity. Cell 71, 301–313. 10.1016/0092-8674(92)90358-J 1423595

[B59] Gerber A. P. Herschlag D. Brown P. O. (2004). Extensive association of functionally and cytotopically related mRNAs with Puf family RNA-binding proteins in yeast. PLoS Biol. 2:E79. 10.1371/journal.pbio.0020079 15024427 PMC368173

[B60] Gogendeau D. Basto R. (2010). Centrioles in flies: the exception to the rule? Semin. Cell Dev. Biol. 21, 163–173. 10.1016/j.semcdb.2009.07.001 19596460

[B61] Gramates L. S. Marygold S. J. Santos G. D. Urbano J. M. Antonazzo G. Matthews B. B.. (2017). FlyBase at 25: looking to the future. Nucleic Acids Res. 45, D663–D671. 10.1093/nar/gkw1016 27799470 PMC5210523

[B62] Greenspan R. J. (2003). RNA and memory: from feeding to localization. Curr. Biol. 13, R126–R127. 10.1016/S0960-9822(03)00071-X 12593811

[B63] Greiss F. Deligiannaki M. Jung C. Gaul U. Braun D. (2016). Single-molecule imaging in living *Drosophila* embryos with reflected light-sheet microscopy. Biophys. J. 110, 939–946. 10.1016/j.bpj.2015.12.035 26910430 PMC4776035

[B64] Guruharsha K. G. Rual J. F. Zhai B. Mintseris J. Vaidya P. Vaidya N.. (2011). A protein complex network of *Drosophila melanogaster*. Cell 147, 690–703. 10.1016/j.cell.2011.08.047 22036573 PMC3319048

[B65] Gustavsson A. K. Petrov P. N. Lee M. Y. Shechtman Y. Moerner W. E. (2018). 3D single-molecule super-resolution microscopy with a tilted light sheet. Nat. Commun. 9:123. 10.1038/s41467-017-02563-4 29317629 PMC5760554

[B66] Habermann K. Lange B. M. (2012). New insights into subcomplex assembly and modifications of centrosomal proteins. Cell Div. 7:17. 10.1186/1747-1028-7-17 22800182 PMC3479078

[B67] Hafen E. Levine M. Garber R. L. Gehring W. J. (1983). An improved *in situ* hybridization method for the detection of cellular RNAs in *Drosophila* tissue sections and its application for localizing transcripts of the homeotic Antennapedia gene complex. EMBO J. 2, 617–623. 10.1002/j.1460-2075.1983.tb01472.x 16453446 PMC555070

[B68] Haimovich G. Cohen-Zontag O. Gerst J. E. (2016a). A role for mRNA trafficking and localized translation in peroxisome biogenesis and function? Biochim. Biophys. Acta 1863, 911–921. 10.1016/j.bbamcr.2015.09.007 26367800

[B69] Haimovich G. Zabezhinsky D. Haas B. Slobodin B. Purushothaman P. Fan L.. (2016b). Use of the MS2 aptamer and coat protein for RNA localization in yeast: a response to “MS2 coat proteins bound to yeast mRNAs block 5' to 3' degradation and trap mRNA decay products: implications for the localization of mRNAs by MS2-MCP system”. RNA 22, 660–666. 10.1261/rna.055095.115 26968626 PMC4836641

[B70] Halstead J. M. Lionnet T. Wilbertz J. H. Wippich F. Ephrussi A. Singer R. H.. (2015). Translation. An RNA biosensor for imaging the first round of translation from single cells to living animals. Science 347, 1367–1671. 10.1126/science.aaa3380 25792328 PMC4451088

[B71] Hartenstein V. Campos-Ortega J. A. (1984). Early neurogenesis in wild-type*Drosophila melanogaster*. Wilehm Roux. Arch. Dev. Biol. 193, 308–325. 10.1007/BF00848159 28305340

[B72] Hartman H. Puma J. P. Gruney T. (1974). Evidence for the association of RNA with the ciliary basal bodies of Tetrahymena. J. Cell Sci. 16, 241–259. 4217340 10.1242/jcs.16.2.241

[B73] Hayashi R. Wainwright S. M. Liddell S. J. Pinchin S. M. Horswell S. Ish-Horowicz D. (2014). A genetic screen based on *in vivo* RNA imaging reveals centrosome-independent mechanisms for localizing gurken transcripts in *Drosophila*. G3 (Bethesda). 4, 749–760. 10.1534/g3.114.010462 24531791 PMC4059244

[B74] Heidemann S. R. Sander G. Kirschner M. W. (1977). Evidence for a functional role of RNA in centrioles. Cell 10, 337–350. 10.1016/0092-8674(77)90021-6 403009

[B75] Heinrich S. Derrer C. P. Lari A. Weis K. Montpetit B. (2017a). Temporal and spatial regulation of mRNA export: single particle RNA-imaging provides new tools and insights. Bioessays 39:1600124. 10.1002/bies.201600124 28052353 PMC5992323

[B76] Heinrich S. Sidler C. L. Azzalin C. M. Weis K. (2017b). Stem–loop RNA labeling can affect nuclear and cytoplasmic mRNA processing. RNA 23, 134–141. 10.1261/rna.057786.116 28096443 PMC5238788

[B77] Heisenberg M. (2003). Mushroom body memoir: from maps to models. Nat. Rev. Neurosci. 4, 266–275. 10.1038/nrn1074 12671643

[B78] Heisenberg M. Borst A. Wagner S. Byers D. (1985). *Drosophila* mushroom body mutants are deficient in olfactory learning. J. Neurogenet. 2, 1–30. 10.3109/01677068509100140 4020527

[B79] Heraud-Farlow J. E. Kiebler M. A. (2014). The multifunctional Staufen proteins: conserved roles from neurogenesis to synaptic plasticity. Trends Neurosci. 37, 470–479. 10.1016/j.tins.2014.05.009 25012293 PMC4156307

[B80] Hermesh O. Jansen R. P. (2013). Take the (RN)A-train: localization of mRNA to the endoplasmic reticulum. Biochim. Biophys. Acta 1833, 2519–2525. 10.1016/j.bbamcr.2013.01.013 23353632

[B81] Hirata J. Nakagoshi H. Nabeshima Y. Matsuzaki F. (1995). Asymmetric segregation of the homeodomain protein prospero during *Drosophila* development. Nature 377, 627–630. 10.1038/377627a0 7566173

[B82] Homem C. C. Knoblich J. A. (2012). *Drosophila* neuroblasts: a model for stem cell biology. Development 139, 4297–4310. 10.1242/dev.080515 23132240

[B83] Hong Y. Stronach B. Perrimon N. Jan L. Y. Jan Y. N. (2001). *Drosophila* stardust interacts with crumbs to control polarity of epithelia but not neuroblasts. Nature 414, 634–638. 10.1038/414634a 11740559

[B84] Hörnberg H. Holt C. (2013). RNA-binding proteins and translational regulation in axons and growth cones. Front. Neurosci. 7:81. 10.3389/fnins.2013.00081 23734093 PMC3661996

[B85] Horne-Badovinac S. Bilder D. (2008). Dynein regulates epithelial polarity and the apical localization of stardust a mRNA. PLoS Genet. 4:e8. 10.1371/journal.pgen.0040008 18208331 PMC2213700

[B86] Hughes J. R. Bullock S. L. Ish-Horowicz D. (2004). Inscuteable mRNA localization is dynein-dependent and regulates apicobasal polarity and spindle length in *Drosophila* neuroblasts. Curr. Biol. 14, 1950–1956. 10.1016/j.cub.2004.10.022 15530398

[B87] Hughes S. C. Krause H. M. (1998). Double labeling with fluorescence *in situ* hybridization in *Drosophila* whole-mount embryos. BioTechniques 24, 530–532. 10.2144/98244bm01 9564514

[B88] Hughes S. C. Krause H. M. (1999). Single and double FISH protocols for *Drosophila*. Methods Mol. Biol. 122, 93–101. 10231786 10.1385/1-59259-722-x:93

[B89] Hughes S. C. Saulier-Le Drean B. Livne-Bar I. Krause H. M. (1996). Fluorescence *in situ* hybridization in whole-mount *Drosophila* embryos. BioTechniques 20, 748–750. 10.2144/96205bm01 8723908

[B90] Hutterer A. Betschinger J. Petronczki M. Knoblich J. A. (2004). Sequential roles of Cdc42, Par-6, aPKC, and Lgl in the establishment of epithelial polarity during *Drosophila* embryogenesis. Dev. Cell 6, 845–854. 10.1016/j.devcel.2004.05.003 15177032

[B91] Irion U. Leptin M. Siller K. Fuerstenberg S. Cai Y. Doe C. Q.. (2004). Abstrakt, a DEAD box protein, regulates Insc levels and asymmetric division of neural and mesodermal progenitors. Curr. Biol. 14, 138–144. 10.1016/j.cub.2004.01.002 14738736

[B92] Jambor H. Surendranath V. Kalinka A. T. Mejstrik P. Saalfeld S. Tomancak P. (2015). Systematic imaging reveals features and changing localization of mRNAs in *Drosophila* development. eLife 4:e05003. 10.7554/eLife.05003 25838129 PMC4384636

[B93] Jandura A. Hu J. Wilk R. Krause H. M. (2017). High resolution fluorescent *in situ* hybridization in *Drosophila* embryos and tissues using tyramide signal amplification. J. Vis. Exp. 128:e56281 10.3791/56281 PMC5752429 29155736

[B94] Jiang N. Kim H. J. Chozinski T. J. Azpurua J. E. Eaton B. A. Vaughan J. C.. (2018). Superresolution imaging of *Drosophila* tissues using expansion microscopy. Mol. Biol. Cell 29, 1413–1421. 10.1091/mbc.E17-10-0583 29688792 PMC6014096

[B95] Jung H. Yoon B. C. Holt C. E. (2012). Axonal mRNA localization and local protein synthesis in nervous system assembly, maintenance and repair. Nat. Rev. Neurosci. 13, 308–324. 10.1038/nrn3210 22498899 PMC3682205

[B96] Keefe A. C. Kardon G. (2015). A new role for dystrophin in muscle stem cells. Nat. Med. 21:1391. 10.1038/nm.4006 26646493

[B97] Keshishian H. Broadie K. Chiba A. Bate M. (1996). The *Drosophila* neuromuscular junction: a model system for studying synaptic development and function. Annu. Rev. Neurosci. 19, 545–575. 10.1146/annurev.ne.19.030196.002553 8833454

[B98] Kindler S. Kreienkamp H. J. (2012). Dendritic mRNA targeting and translation. Adv. Exp. Med. Biol. 970, 285–305. 10.1007/978-3-7091-0932-8_13 22351061

[B99] Kingsley E. P. Chan X. Y. Duan Y. Lambert J. D. (2007). Widespread RNA segregation in a spiralian embryo. Evol. Dev. 9, 527–539. 10.1111/j.1525-142X.2007.00194.x 17976050

[B100] Knoblich J. A. Jan L. Y. Jan Y. N. (1995). Asymmetric segregation of numb and prospero during cell division. Nature 377, 624–627. 10.1038/377624a0 7566172

[B101] Kramer S. (2017). Simultaneous detection of mRNA transcription and decay intermediates by dual colour single mRNA FISH on subcellular resolution. Nucleic Acids Res. 45:e49. 10.1093/nar/gkw1245 27940558 PMC5397161

[B102] Krestel H. Meier J. C. (2018). RNA editing and retrotransposons in neurology. Front. Mol. Neurosci. 11:163. 10.3389/fnmol.2018.00163 29875629 PMC5974252

[B103] Kristó I. Bajusz C. Borsos B. N. Pankotai T. Dopie J. Jankovics F.. (2017). The actin binding cytoskeletal protein Moesin is involved in nuclear mRNA export. Biochim. Biophys. Acta 1864, 1589–1604. 10.1016/j.bbamcr.2017.05.020 28554770

[B104] Kuchinke U. Grawe F. Knust E. (1998). Control of spindle orientation in *Drosophila* by the Par-3-related PDZ-domain protein Bazooka. Curr. Biol. 8, 1357–1365. 10.1016/S0960-9822(98)00016-5 9889099

[B105] Lajeunesse D. R. McCartney B. M. Fehon R. G. (1998). Structural analysis of *Drosophila* Merlin reveals functional domains important for growth control and subcellular localization. J. Cell Biol. 141, 1589–1599. 10.1083/jcb.141.7.1589 9647651 PMC2133006

[B106] Lambert J. D. Nagy L. M. (2002). Asymmetric inheritance of centrosomally localized mRNAs during embryonic cleavages. Nature 420, 682–686. 10.1038/nature01241 12478296

[B107] Lantz V. Schedl P. (1994). Multiple cis-acting targeting sequences are required for orb mRNA localization during *Drosophila* oogenesis. Mol. Cell. Biol. 14, 2235–2242. 10.1128/MCB.14.4.2235 8139529 PMC358590

[B108] Lasko P. (2011). Posttranscriptional regulation in *Drosophila* oocytes and early embryos. Wiley Interdiscip. Rev. RNA 2, 408–416. 10.1002/wrna.70 21957026

[B109] Lasko P. (2012). mRNA localization and translational control in *Drosophila* oogenesis. Cold Spring Harb. Perspect. Biol. 4:a012294. 10.1101/cshperspect.a012294 22865893 PMC3475173

[B110] Laver J. D. Marsolais A. J. Smibert C. A. Lipshitz H. D. (2015). Regulation and function of maternal gene products during the maternal-to-zygotic transition in *Drosophila*. Curr. Top. Dev. Biol. 113, 43–84. 10.1016/bs.ctdb.2015.06.007 26358870

[B111] Lécuyer E. Parthasarathy N. Krause H. M. (2008). Fluorescent *in situ* hybridization protocols in *Drosophila* embryos and tissues. Methods Mol. Biol. 420, 289–302. 10.1007/978-1-59745-583-1_18 18641955

[B112] Lee C. Le M. P. Wallingford J. B. (2009). The Shroom family proteins play broad roles in the morphogenesis of thickened epithelial sheets. Dev. Dyn. 238, 1480–1491. 10.1002/dvdy.21942 19384856 PMC2699254

[B113] Lee H. Y. Haurwitz R. E. Apffel A. Zhou K. Smart B. Wenger C. D.. (2013). RNA–protein analysis using a conditional CRISPR nuclease. Proc. Natl. Acad. Sci. U.S.A. 110, 5416–5421. 10.1073/pnas.1302807110 23493562 PMC3619310

[B114] Lee T. Lee A. Luo L. (1999). Development of the *Drosophila* mushroom bodies: sequential generation of three distinct types of neurons from a neuroblast. Development 126, 4065–4076. 10457015 10.1242/dev.126.18.4065

[B115] Levine M. Hafen E. Garber R. L. Gehring W. J. (1983). Spatial distribution of Antennapedia transcripts during *Drosophila* development. EMBO J. 2, 2037–2046. 10.1002/j.1460-2075.1983.tb01697.x 6416828 PMC555406

[B116] Li P. Yang X. Wasser M. Cai Y. Chia W. (1997). Inscuteable and staufen mediate asymmetric localization and segregation of prospero RNA during *Drosophila* neuroblast cell divisions. Cell 90, 437–447. 10.1016/S0092-8674(00)80504-8 9267024

[B117] Li Z. Wang L. Hays T. S. Cai Y. (2008). Dynein-mediated apical localization of crumbs transcripts is required for Crumbs activity in epithelial polarity. J. Cell Biol. 180, 31–38. 10.1083/jcb.200707007 18195099 PMC2213619

[B118] Little S. C. Gregor T. (2018). Single mRNA molecule detection in *Drosophila*. Methods Mol. Biol. 1649, 127–142. 10.1007/978-1-4939-7213-5_8 29130194 PMC5772605

[B119] Maas S. (2012). Posttranscriptional recoding by RNA editing. Adv. Protein Chem. Struct. Biol. 86, 193–224. 10.1016/B978-0-12-386497-0.00006-2 22243585

[B120] Macdonald P. M. Struhl G. (1988). *Cis*-acting sequences responsible for anterior localization of *bicoid* mRNA in *Drosophila* embryos. Nature 336, 595–598. 10.1038/336595a0 3143913

[B121] Mach J. M. Lehmann R. (1997). An Egalitarian-BicaudalD complex is essential for oocyte specification and axis determination in *Drosophila*. Genes Dev. 11, 423–435. 10.1101/gad.11.4.423 9042857

[B122] Mahen R. Venkitaraman A. R. (2012). Pattern formation in centrosome assembly. Curr. Opin. Cell Biol. 24, 14–23. 10.1016/j.ceb.2011.12.012 22245706

[B123] Martin K. C. Ephrussi A. (2009). mRNA localization: gene expression in the spatial dimension. Cell 136, 719–730. 10.1016/j.cell.2009.01.044 19239891 PMC2819924

[B124] Mast F. D. Li J. Virk M. K. Hughes S. C. Simmonds A. J. Rachubinski R. A. (2011). A *Drosophila* model for the Zellweger spectrum of peroxisome biogenesis disorders. Dis. Model. Mech. 4, 659–672. 10.1242/dmm.007419 21669930 PMC3180231

[B125] Matsuzaki F. Ohshiro T. Ikeshima-Kataoka H. Izumi H. (1998). miranda localizes staufen and prospero asymmetrically in mitotic neuroblasts and epithelial cells in early *Drosophila* embryogenesis. Development 125, 4089–4098. 9735369 10.1242/dev.125.20.4089

[B126] McCartney B. M. Kulikauskas R. M. Lajeunesse D. R. Fehon R. G. (2000). The Neurofibromatosis-2 homologue, Merlin, and the tumor suppressor expanded function together in *Drosophila* to regulate cell proliferation and differentiation. Development 127, 1315–1324. 10683183 10.1242/dev.127.6.1315

[B127] Médina E. Williams J. Klipfell E. Zarnescu D. Thomas G. Le Bivic A. (2002). Crumbs interacts with moesin and beta(Heavy)-spectrin in the apical membrane skeleton of *Drosophila*. J. Cell Biol. 158, 941–951. 10.1083/jcb.200203080 12213838 PMC2173152

[B128] Medioni C. Mowry K. Besse F. (2012). Principles and roles of mRNA localization in animal development. Development 139, 3263–3276. 10.1242/dev.078626 22912410 PMC3424039

[B129] Medioni C. Ramialison M. Ephrussi A. Besse F. (2014). Imp promotes axonal remodeling by regulating profilin mRNA during brain development. Curr. Biol. 24, 793–800. 10.1016/j.cub.2014.02.038 24656828

[B130] Meier J. C. Kankowski S. Krestel H. Hetsch F. (2016). RNA editing—systemic relevance and clue to disease mechanisms? Front. Mol. Neurosci. 9:124. 10.3389/fnmol.2016.00124 27932948 PMC5120146

[B131] Menon K. P. Carrillo R. A. Zinn K. (2013). Development and plasticity of the *Drosophila* larval neuromuscular junction. Wiley Interdiscip. Rev. Dev. Biol. 2, 647–670. 10.1002/wdev.108 24014452 PMC3767937

[B132] Misra M. Edmund H. Ennis D. Schlueter M. A. Marot J. E. Tambasco J.. (2016). A genome-wide screen for dendritically localized RNAs identifies genes required for dendrite morphogenesis. G3 (Bethesda). 6, 2397–2405. 10.1534/g3.116.030353 27260999 PMC4978894

[B133] Morisaki T. Lyon K. Deluca K. F. Deluca J. G. English B. P. Zhang Z.. (2016). Real-time quantification of single RNA translation dynamics in living cells. Science 352, 1425–1429. 10.1126/science.aaf0899 27313040

[B134] Mosca T. J. Luginbuhl D. J. Wang I. E. Luo L. (2017). Presynaptic LRP4 promotes synapse number and function of excitatory CNS neurons. Elife 6:e27347. 10.7554/eLife.27347 28606304 PMC5469616

[B135] Moyne G. Garrido J. (1976). Ultrastructural evidence of mitotic perichromosomal ribonucleoproteins in hamster cells. Exp. Cell Res. 98, 237–247. 10.1016/0014-4827(76)90433-X 943298

[B136] Muller H. Schmidt D. Steinbrink S. Mirgorodskaya E. Lehmann V. Habermann K.. (2010). Proteomic and functional analysis of the mitotic *Drosophila* centrosome. EMBO J. 29, 3344–3357. 10.1038/emboj.2010.210 20818332 PMC2957212

[B137] Najand N. Simmonds A. J. (2007). A minimal WLE2 element is not sufficient to direct apical localization in the absence of RNAs containing the full length *wingless* 3'UTR. RNA Biol. 4, 138–146. 10.4161/rna.4.3.5170 18347436

[B138] Nam S. C. Choi K. W. (2003). Interaction of Par-6 and crumbs complexes is essential for photoreceptor morphogenesis in *Drosophila*. Development 130, 4363–4372. 10.1242/dev.00648 12900452

[B139] Nigg E. A. Stearns T. (2011). The centrosome cycle: centriole biogenesis, duplication and inherent asymmetries. Nat. Cell Biol. 13, 1154–1160. 10.1038/ncb2345 21968988 PMC3947860

[B140] Noh J. H. Kim K. M. Mcclusky W. G. Abdelmohsen K. Gorospe M. (2018). Cytoplasmic functions of long noncoding RNAs. Wiley Interdisc. Rev. RNA 9:e1471. 10.1002/wrna.1471 29516680 PMC5963534

[B141] Olesnicky E. C. Killian D. J. Garcia E. Morton M. C. Rathjen A. R. Sola I. E.. (2014). Extensive use of RNA-binding proteins in *Drosophila* sensory neuron dendrite morphogenesis. G3 (Bethesda). 4, 297–306. 10.1534/g3.113.009795 24347626 PMC3931563

[B142] Olesnicky E. C. Wright E. G. (2018). *Drosophila* as a model for assessing the function of RNA-binding proteins during neurogenesis and neurological disease. J. Dev. Biol. 6:E21. 10.3390/jdb6030021 30126171 PMC6162566

[B143] O'neill J. W. Bier E. (1994). Double-label *in situ* hybridization using biotin and digoxigenin-tagged RNA probes. BioTechniques 17, 870, 874–875. 7840966

[B144] Ota T. Shimamura T. (1956). Cytochemical studies on the mitotic spindle and the phragmoplast of plant cells. Exp. Cell Res. 11, 346–361. 10.1016/0014-4827(56)90111-2 13375657

[B145] Packard M. Jokhi V. Ding B. Ruiz-Canada C. Ashley J. Budnik V. (2015). Nucleus to synapse nesprin1 railroad tracks direct synapse maturation through RNA localization. Neuron 86, 1015–1028. 10.1016/j.neuron.2015.04.006 25959729 PMC4657559

[B146] Paige J. S. Wu K. Y. Jaffrey S. R. (2011). RNA mimics of green fluorescent protein. Science 333, 642–646. 10.1126/science.1207339 21798953 PMC3314379

[B147] Patel P. H. Barbee S. A. Blankenship J. T. (2016). GW-bodies and P-bodies constitute two separate pools of sequestered non-translating RNAs. PLoS ONE 11:e0150291. 10.1371/journal.pone.0150291 26930655 PMC4773245

[B148] Peterson S. P. Berns M. W. (1978). Evidence for centriolar region RNA functioning in spindle formation in dividing PTK2 cells. J. Cell Sci. 34, 289–301. 748344 10.1242/jcs.34.1.289

[B149] Piccolo L. L. Corona D. Onorati M. C. (2014). Emerging roles for hnRNPs in post-transcriptional regulation: what can we learn from flies? Chromosoma 123, 515–527. 10.1007/s00412-014-0470-0 24913828

[B150] Pichon X. Bastide A. Safieddine A. Chouaib R. Samacoits A. Basyuk E.. (2016). Visualization of single endogenous polysomes reveals the dynamics of translation in live human cells. J. Cell Biol. 214, 769–781. 10.1083/jcb.201605024 27597760 PMC5021097

[B151] Piper M. Holt C. (2004). RNA translation in axons. Annu. Rev. Cell Dev. Biol. 20, 505–523. 10.1146/annurev.cellbio.20.010403.111746 15473850 PMC3682640

[B152] Planchon T. A. Gao L. Milkie D. E. Davidson M. W. Galbraith J. A. Galbraith C. G.. (2011). Rapid three-dimensional isotropic imaging of living cells using Bessel beam plane illumination. Nat. Methods 8, 417–423. 10.1038/nmeth.1586 21378978 PMC3626440

[B153] Puthanveettil S. V. (2013). RNA transport and long-term memory storage. RNA Biol. 10, 1765–1770. 10.4161/rna.27391 24356491 PMC3917979

[B154] Putnam A. A. Jankowsky E. (2013). DEAD-box helicases as integrators of RNA, nucleotide and protein binding. Biochim. Biophys. Acta 1829, 884–893. 10.1016/j.bbagrm.2013.02.002 23416748 PMC3661757

[B155] Raff J. W. Whitfield W. G. Glover D. M. (1990). Two distinct mechanisms localise cyclin B transcripts in syncytial *Drosophila* embryos. Development 110, 1249–1261. 2151612 10.1242/dev.110.4.1249

[B156] Ramat A. Hannaford M. Januschke J. (2017). Maintenance of miranda localization in *Drosophila* neuroblasts involves interaction with the cognate mRNA. Curr Biol 27, 2101–2111 e2105. 10.1016/j.cub.2017.06.016 28690114 PMC5526833

[B157] Raut S. Mallik B. Parichha A. Amrutha V. Sahi C. Kumar V. (2017). RNAi-mediated reverse genetic screen identified *Drosophila* chaperones regulating eye and neuromuscular junction morphology. G3 (Bethesda). 7, 2023–2038. 10.1534/g3.117.041632 28500055 PMC5499113

[B158] Rieder C. L. (1979). Localization of ribonucleoprotein in the trilaminar kinetochore of PtK1. J. Ultrastruct. Res. 66, 109–119. 10.1016/S0022-5320(79)90128-X 85718

[B159] Rodal A. A. Del Signore S. J. Martin A. C. (2015). *Drosophila* comes of age as a model system for understanding the function of cytoskeletal proteins in cells, tissues, and organisms. Cytoskeleton 72, 207–224. 10.1002/cm.21228 26074334 PMC4782189

[B160] Rodriguez A. J. Shenoy S. M. Singer R. H. Condeelis J. (2006). Visualization of mRNA translation in living cells. J. Cell Biol. 175:67. 10.1083/jcb.200512137 17030983 PMC2064499

[B161] Rodriguez-Boulan E. Powell S. K. (1992). Polarity of epithelial and neuronal cells. Annu. Rev. Cell Biol. 8, 395–427. 10.1146/annurev.cb.08.110192.002143 1476804

[B162] Rongo C. Gavis E. R. Lehmann R. (1995). Localization of *oskar* RNA regulates *oskar* translation and requires Oskar protein. Development 121, 2737–2746. 7555702 10.1242/dev.121.9.2737

[B163] Rosenthal J. J. (2015). The emerging role of RNA editing in plasticity. J. Exp. Biol. 218, 1812–1821. 10.1242/jeb.119065 26085659 PMC4487009

[B164] Rustad R. C. (1959). An interference microscopical and cytochemical analysis of local mass changes in the mitotic apparatus during mitosis. Exp. Cell Res. 16, 575–583. 10.1016/0014-4827(59)90125-9 13653025

[B165] Sahoo P. K. Smith D. S. Perrone-Bizzozero N. Twiss J. L. (2018). Axonal mRNA transport and translation at a glance. J. Cell Sci. 131:jcs196808. 10.1242/jcs.196808 29654160 PMC6518334

[B166] Schmid M. Jensen T. H. (2018). Controlling nuclear RNA levels. Nat. Rev. Genet. 19:1. 10.1038/s41576-018-0013-2 29748575

[B167] Schuldt A. J. Adams J. H. Davidson C. M. Micklem D. R. Haseloff J. St. Johnston D.. (1998). Miranda mediates asymmetric protein and RNA localization in the developing nervous system. Genes Dev. 12, 1847–1857. 10.1101/gad.12.12.1847 9637686 PMC316902

[B168] Seimetz J. Arif W. Bangru S. Hernaez M. Kalsotra A. (2018). Cell-type specific polysome profiling from mammalian tissues. Methods. 115, 131–139. 10.1016/j.ymeth.2018.11.015 PMC6387852 30500367

[B169] Sen A. Sun R. Krahn M. P. (2015). Localization and function of Pals1-associated tight junction protein in *Drosophila* is regulated by two distinct apical complexes. J. Biol. Chem. 290, 13224–13233. 10.1074/jbc.M114.629014 25847234 PMC4505576

[B170] Sezen B. Seedorf M. Schiebel E. (2009). The SESA network links duplication of the yeast centrosome with the protein translation machinery. Genes Dev. 23, 1559–1570. 10.1101/gad.524209 19571182 PMC2704472

[B171] Shah S. Lubeck E. Schwarzkopf M. He T. F. Greenbaum A. Sohn C. H.. (2016). Single-molecule RNA detection at depth by hybridization chain reaction and tissue hydrogel embedding and clearing. Development 143, 2862–2867. 10.1242/dev.138560 27342713 PMC5004914

[B172] Simmonds A. J. Dossantos G. Livne-Bar I. Krause H. M. (2001). Apical localization of *wingless* transcripts is required for wingless signaling. Cell 105, 197–207. 10.1016/S0092-8674(01)00311-7 11336670

[B173] Singer R. H. Ward D. C. (1982). Actin gene expression visualized in chicken muscle tissue culture by using *in situ* hybridization with a biotinated nucleotide analog. Proc. Natl. Acad. Sci. U.S.A. 79, 7331–7335. 10.1073/pnas.79.23.7331 6961411 PMC347333

[B174] Smith J. J. Aitchison J. D. (2013). Peroxisomes take shape. Nat. Rev. Mol. Cell Biol. 14, 803–817. 10.1038/nrm3700 24263361 PMC4060825

[B175] Smyth J. T. Schoborg T. A. Bergman Z. J. Riggs B. Rusan N. M. (2015). Proper symmetric and asymmetric endoplasmic reticulum partitioning requires astral microtubules. Open. Biol. 5:150067. 10.1098/rsob.150067 26289801 PMC4554919

[B176] Snyder J. A. (1980). Evidence for a ribonucleoprotein complex as a template for microtubule initiation *in vivo*. Cell Biol. Int. Rep. 4, 859–868. 10.1016/0309-1651(80)90184-8 7418015

[B177] Solinet S. Mahmud K. Stewman S. F. Ben El Kadhi K. Decelle B. Talje L.. (2013). The actin-binding ERM protein Moesin binds to and stabilizes microtubules at the cell cortex. J. Cell Biol. 202, 251–260. 10.1083/jcb.201304052 23857773 PMC3718980

[B178] Song Y. Fee L. Lee T. H. Wharton R. P. (2007). The molecular chaperone Hsp90 is required for mRNA localization in *Drosophila melanogaster* embryos. Genetics 176, 2213–2222. 10.1534/genetics.107.071472 17565952 PMC1950626

[B179] Standart N. Weil D. (2018). P-bodies: cytosolic droplets for coordinated mRNA storage. Trends Genet. 34, 612–626. 10.1016/j.tig.2018.05.005 29908710

[B180] Stapleton M. Carlson J. W. Celniker S. E. (2006). RNA editing in *Drosophila melanogaster*: new targets and functional consequences. RNA 12, 1922–1932. 10.1261/rna.254306 17018572 PMC1624909

[B181] Stich H. (1954). Substances and striations in the spindle of Cyclops strenuus; mechanism of mitosis. Chromosoma 6, 199–236. 10.1007/BF01259940 13172850

[B182] St. Johnston R. D. Beuchle D. Nusslein-Volhard C. (1991). staufen, a gene required to localize maternal RNAs in the *Drosophila* egg. Cell 66, 51–63. 10.1016/0092-8674(91)90138-O 1712672

[B183] Subramaniam K. Seydoux G. (1999). nos-1 and nos-2, two genes related to *Drosophila* nanos, regulate primordial germ cell development and survival in Caenorhabditis elegans. Development 126:4861. 10518502 10.1242/dev.126.21.4861

[B184] Supek F. Bošnjak M. Škunca N. Šmuc T. (2011). REVIGO summarizes and visualizes long lists of gene ontology terms. PLoS ONE 6:e21800. 10.1371/journal.pone.0021800 21789182 PMC3138752

[B185] Tautz D. Pfeifle C. (1989). A non-radioactive *in situ* hybridization method for the localization of specific RNAs in *Drosophila* embryos reveals translational control of the segmentation gene hunchback. Chromosoma 98, 81–85. 10.1007/BF00291041 2476281

[B186] Tepass U. (2012). The apical polarity protein network in *Drosophila* epithelial cells: regulation of polarity, junctions, morphogenesis, cell growth, and survival. Annu. Rev. Cell Dev. Biol. 28, 655–685. 10.1146/annurev-cellbio-092910-154033 22881460

[B187] Tepass U. Tanentzapf G. Ward R. Fehon R. (2001). Epithelial cell polarity and cell junctions in *Drosophila*. Annu. Rev. Genet. 35, 747–784. 10.1146/annurev.genet.35.102401.091415 11700298

[B188] Tepass U. Theres C. Knust E. (1990). *Crumbs* encodes an EGF-like protein expressed on apical membranes of *Drosophila* epithelial cells and required for organization of epithelia. Cell 61, 787–799. 10.1016/0092-8674(90)90189-L 2344615

[B189] Titlow J. S. Yang L. Parton R. M. Palanca A. Davis I. (2018). Super-resolution single molecule FISH at the *Drosophila* neuromuscular junction. Methods Mol. Biol. 1649, 163–175. 10.1007/978-1-4939-7213-5_10 29130196 PMC6128253

[B190] Tom Dieck S. Kochen L. Hanus C. Heumüller M. Bartnik I. Nassim-Assir B.. (2015). Direct visualization of newly synthesized target proteins *in situ*. Nat. Methods 12:411. 10.1038/nmeth.3319 25775042 PMC4414919

[B191] Towler B. P. Newbury S. F. (2018). Regulation of cytoplasmic RNA stability: lessons from *Drosophila*. Wiley Interdisc. Rev. RNA 9:e1499. 10.1002/wrna.1499 30109918

[B192] Trcek T. Rahman S. Zenklusen D. (2018). Measuring mRNA decay in budding yeast using single molecule FISH. Methods Mol. Biol. 1720, 35–54. 10.1007/978-1-4939-7540-2_4 29236250 PMC10773531

[B193] Tsuda M. Sasaoka Y. Kiso M. Abe K. Haraguchi S. Kobayashi S.. (2003). Conserved role of nanos proteins in germ Cell development. Science 301:1239. 10.1126/science.1085222 12947200

[B194] Tutucci E. Vera M. Biswas J. Garcia J. Parker R. Singer R. H. (2018). An improved MS2 system for accurate reporting of the mRNA life cycle. Nat. Methods 15, 81–89. 10.1038/nmeth.4502 29131164 PMC5843578

[B195] Tweedie S. Ashburner M. Falls K. Leyland P. Mcquilton P. Marygold S.. (2009). FlyBase: enhancing *Drosophila* Gene Ontology annotations. Nucleic Acids Res. 37, D555–D559. 10.1093/nar/gkn788 18948289 PMC2686450

[B196] Unger E. (1977). RNA in the spindle pole body of yeasts. Z. Allg. Mikrobiol. 17, 487–490. 10.1002/jobm.3630170610 337693

[B197] Vazquez-Pianzola P. Suter B. (2012). Conservation of the RNA transport machineries and their coupling to translation control across *Eukaryotes*. Comp. Funct. Genomics 2012:287852. 10.1155/2012/287852 22666086 PMC3361156

[B198] Veeranan-Karmegam R. Boggupalli D. P. Liu G. Gonsalvez G. B. (2016). A new isoform of *Drosophila* non-muscle Tropomyosin 1 interacts with Kinesin-1 and functions in oskar mRNA localization. J. Cell Sci. 129, 4252–4264. 10.1242/jcs.194332 27802167 PMC5117202

[B199] Volpe T. Schramke V. Hamilton G. L. White S. A. Teng G. Martienssen R. A.. (2003). RNA interference is required for normal centromere function in fission yeast. Chromosome Res. 11, 137–146. 10.1023/A:1022815931524 12733640

[B200] Wang C. Han B. Zhou R. Zhuang X. (2016). Real-time imaging of translation on single mRNA transcripts in live cells. Cell 165, 990–1001. 10.1016/j.cell.2016.04.040 27153499 PMC4905760

[B201] Watts R. J. Hoopfer E. D. Luo L. (2003). Axon pruning during *Drosophila* metamorphosis: evidence for local degeneration and requirement of the ubiquitin-proteasome system. Neuron 38, 871–885. 10.1016/S0896-6273(03)00295-2 12818174

[B202] Wegener M. Müller-Mcnicoll M. (2018). Nuclear retention of mRNAs - quality control, gene regulation and human disease. Semin. Cell Dev. Biol. 79, 131–142. 10.1016/j.semcdb.2017.11.001 29102717

[B203] Wei W. Ji X. Guo X. Ji S. (2017). Regulatory role of N(6) -methyladenosine (m(6) A) methylation in RNA processing and human diseases. J. Cell. Biochem. 118, 2534–2543. 10.1002/jcb.25967 28256005

[B204] Weigand J. E. Suess B. (2009). Aptamers and riboswitches: perspectives in biotechnology. Appl. Microbiol. Biotechnol. 85, 229–236. 10.1007/s00253-009-2194-2 19756582

[B205] Weil T. T. (2014). mRNA localization in the *Drosophila* germline. RNA Biol. 11, 1010–1018. 10.4161/rna.36097 25482896 PMC4615827

[B206] Weil T. T. (2015). Post-transcriptional regulation of early embryogenesis. F1000Prime Rep. 7:31. 10.12703/P7-31 25926982 PMC4371236

[B207] Weis B. L. Schleiff E. Zerges W. (2013). Protein targeting to subcellular organelles via MRNA localization. Biochim. Biophys. Acta 1833, 260–273. 10.1016/j.bbamcr.2012.04.004 23457718

[B208] Wilk R. Hu J. Blotsky D. Krause H. M. (2016). Diverse and pervasive subcellular distributions for both coding and long noncoding RNAs. Genes Dev. 30, 594–609. 10.1101/gad.276931.115 26944682 PMC4782052

[B209] Wilk R. Hu J. Krause H. M. (2013). Spatial profiling of nuclear receptor transcription patterns over the course of *Drosophila* development. G3 (Bethesda). 3, 1177–1189. 10.1534/g3.113.006023 23665880 PMC3704245

[B210] Wilk R. Murthy S. U. M. Yan H. Krause H. M. (2010). *In situ* hybridization: fruit fly embryos and tissues. Curr. Protoc. Essent. Lab. Tech. 4, 9.3.1–9.3.24. 10.1002/9780470089941.et0903s04

[B211] Wilkie G. S. Davis I. (2001). *Drosophila wingless* and pair-rule transcripts localize apically by dynein-mediated transport of RNA particles. Cell 105, 209–219. 10.1016/S0092-8674(01)00312-9 11336671

[B212] Wu B. Eliscovich C. Yoon Y. J. Singer R. H. (2016). Translation dynamics of single mRNAs in live cells and neurons. Science 352, 1430–1435. 10.1126/science.aaf1084 27313041 PMC4939616

[B213] Xu C. S. Hayworth K. J. Lu Z. Grob P. Hassan A. M. Garcia-Cerdan J. G.. (2017). Enhanced FIB-SEM systems for large-volume 3D imaging. eLife 6:e25916. 10.7554/eLife.25916 28500755 PMC5476429

[B214] Xu X. Brechbiel J. L. Gavis E. R. (2013). Dynein-dependent transport of nanos RNA in *Drosophila* sensory neurons requires Rumpelstiltskin and the germ plasm organizer Oskar. J. Neurosci. 33, 14791–14800. 10.1523/JNEUROSCI.5864-12.2013 24027279 PMC3771026

[B215] Yamashita Y. M. (2018). Subcellular specialization and organelle behavior in germ cells. Genetics 208, 19–51. 10.1534/genetics.117.300184 29301947 PMC5753857

[B216] Yan X. Hoek T. A. Vale R. D. Tanenbaum M. E. (2016). Dynamics of translation of single mRNA molecules *in vivo*. Cell 165, 976–989. 10.1016/j.cell.2016.04.034 27153498 PMC4889334

[B217] Yarmishyn A. A. Kremenskoy M. Batagov A. O. Preuss A. Wong J. H. Kurochkin I. V. (2016). Genome-wide analysis of mRNAs associated with mouse peroxisomes. BMC Genomics 17:1028. 10.1186/s12864-016-3330-x 28155669 PMC5259856

[B218] Ye B. Petritsch C. Clark I. E. Gavis E. R. Jan L. Y. Jan Y. N. (2004). Nanos and Pumilio are essential for dendrite morphogenesis in *Drosophila* peripheral neurons. Curr. Biol. 14, 314–321. 10.1016/j.cub.2004.01.052 14972682

[B219] Yoo S. Van Niekerk E. A. Merianda T. T. Twiss J. L. (2010). Dynamics of axonal mRNA transport and implications for peripheral nerve regeneration. Exp. Neurol. 223, 19–27. 10.1016/j.expneurol.2009.08.011 19699200 PMC2849851

[B220] Zackroff R. V. Rosenfeld A. C. Weisenberg R. C. (1976). Effects of RNase and RNA on *in vitro* aster assembly. J. Supramol. Struct. 5:577589. 10.1002/jss.400050412 1027923

[B221] Zhang T. Tan P. Wang L. Jin N. Li Y. Zhang L.. (2017). RNALocate: a resource for RNA subcellular localizations. Nucleic Acids Res. 45, D135–D138. 10.1093/nar/gkw728 27543076 PMC5210605

[B222] Zimmerman A. M. (1960). Physico-chemical analysis of the isolated mitotic apparatus. Exp. Cell Res. 20, 529–547. 10.1016/0014-4827(60)90122-1 13788525

[B223] Zipor G. Haim-Vilmovsky L. Gelin-Licht R. Gadir N. Brocard C. Gerst J. E. (2009). Localization of mRNAs coding for peroxisomal proteins in the yeast, Saccharomyces cerevisiae. Proc. Natl. Acad. Sci. U.S.A. 106, 19848–19853. 10.1073/pnas.0910754106 19903887 PMC2785255

